# Opportunities for mobilizing recalcitrant phosphorus from agricultural soils: a review

**DOI:** 10.1007/s11104-017-3362-2

**Published:** 2017-08-01

**Authors:** Daniel Menezes-Blackburn, Courtney Giles, Tegan Darch, Timothy S. George, Martin Blackwell, Marc Stutter, Charles Shand, David Lumsdon, Patricia Cooper, Renate Wendler, Lawrie Brown, Danilo S. Almeida, Catherine Wearing, Hao Zhang, Philip M. Haygarth

**Affiliations:** 1 Lancaster Environment Centre, Lancaster University, Lancaster, LA1 4YQ UK; 2 The James Hutton Institute, Dundee and Aberdeen, Scotland DD2 5DA and AB15 8QH UK; 3 Rothamsted Research, North Wyke, Okehampton, Devon EX20 2SB UK; 4 College of Agricultural Sciences, Department of Crop Science, São Paulo State University, Botucatu, 18610-307 Brazil

**Keywords:** Phosphorus, Organic phosphorus, Soil, Crops, Fertilizer, Plant nutrition

## Abstract

**Background:**

Phosphorus (P) fertilizer is usually applied in excess of plant requirement and accumulates in soils due to its strong adsorption, rapid precipitation and immobilisation into unavailable forms including organic moieties. As soils are complex and diverse chemical, biochemical and biological systems, strategies to access recalcitrant soil P are often inefficient, case specific and inconsistently applicable in different soils. Finding a near-universal or at least widely applicable solution to the inefficiency in agricultural P use by plants is an important unsolved problem that has been under investigation for more than half a century.

**Scope:**

In this paper we critically review the strategies proposed for the remobilization of recalcitrant soil phosphorus for crops and pastures worldwide. We have additionally performed a meta-analysis of available soil ^31^P–NMR data to establish the potential agronomic value of different stored P forms in agricultural soils.

**Conclusions:**

Soil inorganic P stocks accounted on average for 1006 ± 115 kg ha^−1^ (57 ± 7%), while the monoester P pool accounted for 587 ± 32 kg ha^−1^ (33 ± 2%), indicating the huge potential for the future agronomic use of the soil legacy P. New impact driven research is needed in order to create solutions for the sustainable management of soil P stocks.

**Electronic supplementary material:**

The online version of this article (doi:10.1007/s11104-017-3362-2) contains supplementary material, which is available to authorized users.

## Introduction

Historically, agricultural strategies to cope with the large phosphorus (P) fixing capacity of many soils have relied on saturating the system with P in the form of fertilizer, derived from non-renewable rock phosphates, to maintain plant-optimum P concentrations in soil solution (Fox and Kamprath 1970). In some countries, long term fertilizer applications to meet plant needs have led to a build-up of a legacy soil P ‘bank’, which is largely unavailable to plants (Kamprath 1967). Recent scientific efforts have been directed toward increasing the plant availability of this legacy soil P and enabling an efficient agronomic use of this important P reserve. But, how likely is legacy soil P to be a key source of P to sustain agricultural production? For how many growing seasons would legacy soil P be able to sustain crop production, and what yields may be expected? What are our most promising and sustainable agroecological innovations to accomplish this?

Modern agricultural dependence on non-renewable natural resources, namely P fertilizers and fossil fuels, is problematic. However, while renewable alternatives to fossil fuels are available, there are still no renewable alternative sources of fertilizer P to rock phosphate mining (Cordell et al. 2009). As rock phosphate mineral resources decline, phosphate fertilizers will inevitably become a scarce, and consequently a costly input, with severe effects on agricultural production and food security (Abelson 1999; Cordell et al. 2009). Additionally, there is the issue of volatility in rock phosphate supply and the related price oscillations, like the recent sharp increase in price which occurred in 2008 with direct impact on the market value of P fertilizers and in the FAO food price index (Cordell and White 2014). Most of the known reserves of rock phosphate are present in Morocco (74%) while Europe has virtually no rock phosphate remaining, and therefore geopolitical issues will be increasingly influential in future P production. Improving P cycling in soils and using recycled P fertilizer sources are not likely to be complete solutions to a future P crisis, but are key contributors to delaying and reducing the impact of a P scarcity scenario (Stutter et al. 2012). Model simulations show that the residual soil P pool may play a role in reducing global P fertilizer use by up to 50% by 2050, in relation to other estimates that do not consider the plant use of soil fixed P (Sattari et al. 2012). Here we argue that an even larger reduction in P fertilizer input could be accomplished if appropriate technologies were applied in mobilizing recalcitrant forms of soil P currently not considered in P use models, and that represent a legacy from historic fertilizer inputs.

## Phosphorus fixation and bioavailability in soils

Phosphorus is perhaps, amongst all the plant nutrients, the one with the most limited bioavailability in soils (Vance et al. 2003). Typically, approximately 6% (range 1.5 to 11%) of total soil P is readily available (Olsen P) while the majority of it is locked in primary minerals, precipitated, adsorbed or in organically-complexed forms (Condron et al. 2005a; Pierzynski et al. 2005; Stutter et al. 2012). To ensure optimal plant growth, phosphate fertilizers are applied to agricultural soils in excess of plant requirements to overcome soil P fixation processes and maintain soil solution P at optimal levels for plant growth (Syers et al. 2008). Long term P fertilizer or P rich manure application is directly proportional to the soil accumulation of up to two thirds of the applied P dose, leading to the progressive saturation of soils with P and the concomitant reduction in P-retention capacity of the soil matrix (Hooda et al. 2001). For example, in Western Europe more than 1.1 tons of P ha^−1^ were applied on average to cropland soils between 1965 and 2007 while less than 0.3 tons ha^−1^ are estimated to have been removed from these systems during the same period (Sattari et al. 2012). Many European soils are excessively fertilized, accumulating soil P pools at levels that are environmentally unacceptable due to the risk of P transfer to watercourses, and the potential for eutrophication (Barberis et al. 1995; Dodd and Sharpley 2015; Haygarth et al. 2014). It is likely this applies to most soils worldwide with a long P fertilizer application history.

Soluble P from freshly applied fertilizer interacts with soil surfaces, displacing other anions with less affinity, to become adsorbed (Pierzynski et al. 2005; Syers et al. 2008). Processes of P sorption and desorption are hysteretic, and desorption rates are much slower than sorption rates at common soil solution P concentrations (Menezes-Blackburn et al. 2016c). Precipitation and surface co-adsorption with metals also play an important role in short term soil P fixation (Hedley and McLaughlin 2005; Li and Stanforth 2000). After fertilizer application, soluble P levels increase to a transient soil solution P concentration, and net P adsorption and precipitation takes place until equilibrium is reached (Fox and Kamprath 1970; Hedley and McLaughlin 2005; Pierzynski et al. 2005). The fixation rates of soluble inorganic P (P_i_) in agricultural soils are usually large and agronomic optimum levels of soil solution P may not be sustained even through one agricultural cycle (Kovar and Claassen 2005; Syers et al. 2008). If fertilizer application is stopped or reduced, solution P is depleted and the equilibrium turns into a slow net solubilisation and desorption of stabilized soil P. The P desorption rate is markedly different between soils of different pH (Smet et al. 1998), and of different mineralogy and organic matter content, and therefore these factors are likely to be key regulators of plant P uptake (Barros and Comerford 2005; Koopmans et al. 2004).

Although plants can only uptake inorganic orthophosphate anions (a component of the inorganic P, P_i_), a considerable fraction (30% to 65%) of soil P is present as organic P forms (P_o_) (Condron et al. 2005b; Turner et al. 2003b). These soil P_o_ forms are produced when plants and microbes take up orthophosphate, immobilising them into organic molecules essential for life (DNA, phospholipids, inositol phosphates, ATP), and which are deposited in soils upon the death of these organisms (Richardson et al. 2005). Similar to P_i_, adsorption and precipitation processes are responsible for stabilizing soil P_o_ that in some soils can build up to 80% of total soil P (Turner et al. 2002). Since plants can only take up inorganic orthophosphate (Raghothama 2005), mobilizing P_o_ forms requires undertaking two steps: first the release of P_o_ from precipitates and adsorption sites; secondly the mineralization of these into plant available P_i_ through the action of phosphatase enzymes (Clarholm et al. 2015; Richardson et al. 2005). The adsorption and release of P_o_ is controlled by similar geochemical constraints to the ones for P_i_, but in some cases such as for phytate, the strength of reactions can be even greater due to the presence of multiple orthophosphate groups and a higher anionic charge density (Yan et al. 2014). Many different enzyme types are involved in soil P_o_ mineralization and these enzymes show considerable differences in catalytic properties, behaviour and efficiency in soils (Menezes-Blackburn et al. 2013). Some plants naturally exude phosphomonoesterases into the rhizosphere in response to P starvation, however these enzymes have, in general, limited or no activity towards recalcitrant forms of P such as phytate (Jakobsen et al. 2005; Menezes-Blackburn et al. 2013). On the other hand, soil microbes express a diverse range of extracellular phosphatase enzymes capable of hydrolysing different soil P_o_ forms (Dick 1994; Konietzny and Greiner 2004; Menezes-Blackburn et al. 2016a; Tapia-Torres et al. 2016). The extracellular microbial phosphatases usually have a short half-life in soil environments, due to inactivation by metal inhibitors, adsorption, proteolysis, pH and ionic strength shifts (George et al. 2006b). When this microbially-mediated dephosphorylation is insufficient to overcome fixation rates, fresh P_o_ forms are stabilized and accumulate in soils as previously discussed. These processes are all regulated by the solubility of P_o_ forms and presence, abundance and function of phosphatases in soil environments (Giles et al. 2016; Menezes-Blackburn et al. 2013). Understanding the complex interrelation of the factors affecting P_o_ mobilization and those affecting enzyme performance in soil environments still represents a huge challenge. Recent projects have been designed to unveil the dynamics of soil rhizosphere microbiome impacts and functions related to soil carbon mineralization (Nuccio et al. 2014; Shi et al. 2014), but so far nothing at a similar level is being performed with regard to soil organic P .

## How much soil phosphorus can potentially be mobilized?

The amount of P that can be mobilized by different strategies is dependent on the abundance and lability of the targeted chemical P species in each soil environment. We have studied soil ^31^P–NMR data from scientific literature (258 different soils from 41 publications) reporting quantitative speciation of orthophosphate, phosphate monoesters and phosphate diesters groups (Table S1). The NMR method is usually performed with soil NaOH-EDTA extracts and examines the chemical structure of alkali soluble P species, which corresponds on average to 55% of the total soil P (mined literature). This is a strong extraction process that does not reflect bioavailable P in soils. To choice of using ^31^P–NMR data in this analysis, in detriment of other methods was to evaluate stocks of different P chemical species, and their potential future sustainable use. To estimate the agronomic value of the soil P, these concentrations were scaled up into total P stocks (kg P ha^−1^) in the first 15 cm depth of soil. Across all samples, the orthophosphate pool accounted for approximately 57% of the NaOH-EDTA extractable total P, while the monoester P pool accounted for approximately 33% (Table 1). By using an approximate P offtake for arable soils and grasslands from Sattari et al. (2016), on average the total P stocks represent 352 ± 26 years’ worth of P for agronomic use; the orthophosphate pool would account for 201 ± 23 years and the monoester pool would account for 117 ± 6 years’ worth of production. This indicates that our strategies for mobilizing soil P for plant nutrition should be focused mainly on the adsorbed and precipitated forms of orthophosphate and on the mineralization of monoester organic P forms like inositol phosphates. The potential of the use of monoester P is slightly greater for grasslands than for arable soils. Large differences in P stocks were also associated with continental distributions (Table 1), with a greater potential use of monoester P in North America, followed by Europe and Oceania; South America, Africa and Asia showed much smaller values, but were excluded from this analysis due to the smaller sample size bringing a stronger bias to this interpretation. There are confounding issues with the analysis of the data in Table 1, including: a) the directed sampling strategy of each individual study; b) insufficient geographical representation; c) differences in the soil extraction efficiency and NMR spectra interpretation; d) samples taken at different times during the last decades and no trends can be found with regard to the dynamics of P accumulation. Although no major analytical inconsistencies are expected when considering ^31^P–NMR data from different sources due to a fairly well standardized approach being adopted, there are potentially minor issues regarding the NaOH-EDTA extraction efficiency, the peak integration method used to interpret the spectra and the choice of equipment setup (delay time, pulse angle, probe size and field strength). Some of these problems were partially overcome by bootstrapping the data with a resample size of 1000, thus decreasing the sample/study specific bias and achieving a better estimation of the population dispersion parameters (Table S2). However, this analysis is only sufficient to demonstrate that there is huge potential to mobilise soil P for future agronomic use.

**Table 1:** Soil phosphorus stocks analysis of global literature on ^31^P–NMR data for agricultural soils. The analysis performed was based on the typical NMR speciation between orthophosphate, monoester P, diester P and other forms of P (phosphonates, pyrophosphate and unidentified P forms) transformed into kg ha^−1^ basis. Values represent the average ± the standard error from Bootstrap analysis (B = 1000; R statistics), and ‘n’ corresponds to the number of soil samples

	Total P	Inorganic Orthophosphate		Monoester		Diester		Other		
	kg ha^−1^	kg ha^−1^	(%)	kg ha^−1^	(%)	kg ha^−1^	(%)	kg ha^−1^	(%)	n
All samples	1762 ± 132	1006 ± 115	(57 ± 7)	587 ± 32	(33 ± 2)	64 ± 7	(4 ± 0)	96 ± 13	(5 ± 1)	258
Arable soils	1666 ± 133	964 ± 72	(58 ± 4)	519 ± 62	(31 ± 4)	64 ± 15	(4 ± 1)	123 ± 28	(7 ± 2)	115
Pastures	1830 ± 220	1037 ± 190	(57 ± 10)	644 ± 28	(35 ± 2)	64 ± 6	(3 ± 0)	74 ± 6	(4 ± 0)	143
Europe	1699 ± 94	927 ± 82	(55 ± 5)	646 ± 28	(38 ± 2)	55 ± 7	(3 ± 0)	68 ± 7	(4 ± 0)	143
North America	2170 ± 327	965 ± 94	(44 ± 4)	842 ± 177	(39 ± 8)	129 ± 42	(6 ± 2)	250 ± 81	(12 ± 4)	35
Oceania	1947 ± 412	1350 ± 363	(69 ± 19)	472 ± 36	(24 ± 2)	44 ± 8	(2 ± 0)	92 ± 14	(5 ± 1)	75

Soil bulk density was used to transform original data from mg kg^−1^ into kg ha^−1^ in the first 15 cm depth. Data was collected from 258 soils and a total of 41 publications (Abdi et al. 2014; Ahlgren et al. 2013; Annaheim et al. 2015; Bourke et al. 2008; Bunemann et al. 2008a, 2008b; Cade-Menun and Preston 1996; Cade-Menun et al. 2010; Chapuis-Lardy et al. 2001; Cheesman et al. 2010; Condron et al. 1990; Doolette et al. 2009, 2010; Doolette et al. 2011; Dougherty et al. 2007; Ebuele et al. 2016; Gatiboni et al. 2007; George et al. 2006a; Giles et al. 2015; Guggenberger et al. 1996a, 1996b; Hill and Cade-Menun 2009; Jin et al. 2016; Koopmans et al. 2003; Lehmann et al. 2005; Leinweber et al. 1997; Liu et al. 2014; McDowell et al. 2005; McDowell and Koopmans 2006; McDowell and Stewart 2006; McLaren et al. 2014, 2015; Moller et al. 2000; Murphy et al. 2009; Soinne et al. 2011; Solomon and Lehman 2000; Solomon et al. 2002; Stutter et al. 2015; Turner 2006; Turner et al. 2003a, 2003b), see Table S1 for detailed information about the data collected and Table S2 for the bootstrapped populations

## Approaches and technologies for sustainably increasing recalcitrant soil phosphorus bioavailability

A sustainable agricultural approach for facing a future rock phosphate shortage should include the unlocking of legacy soil P, in parallel to reducing P fertiliser load, and increasing the use of recycled P sources. The most relevant question is which technologies will ultimately be the most suitable for increasing recalcitrant soil P bioavailability?

The first obvious strategy to increase the use of the soil residual P ‘bank’, involves reducing P fertilizer application rates and allowing adsorbed and precipitated P to restore to equilibrium after P depletion (Menezes-Blackburn et al. 2016c). Nevertheless, this strategy would at some point sacrifice agricultural productivity and it is only suitable for the initial depletion of extremely P rich soils. For most agricultural soils under a depletion scenario, soil solution P levels would decrease below optimal levels for plant growth, and therefore coupled strategies are needed to replenish soil solution P by actively promoting P desorption, solubilisation and mineralization. Sacrificing productivity is unacceptable and incompatible with the need of feeding an ever growing world population. However, in many developed western temperate agriculture systems, due to the increasing prices of P fertilizer the decline in production as a consequence of reducing P inputs may actually improve net returns for producers, where the focus is on profitability rather than maximizing food production. In fact many countries are progressively reducing P fertilizer application rates in response to P sufficiency in soils (Sattari et al. 2012), but not at rates sufficient to undo P fixation, only enough to maintain P fertility and accumulated fixed P at levels capable of sustaining crop productivity.

On the other hand, in many developing countries, mainly in the tropics, P fertilizer inputs have been historically restricted. Conversely, large areas of tropical soils that are increasingly being used for food and animal feed production, now require large P fertilizer inputs. Additionally, the nature of many of these soils will constrain P bioavailability to crops due to their naturally high P fixing characteristics (Richter and Babbar 1991). In these cases, there is often no accumulated P ‘bank’ to exploit. The focus for low P soils such as these is on increasing P fertilizer use efficiency and preventing the accumulation of recalcitrant soil P. Crop rotation using plant species with the ability to scavenge soil recalcitrant P, adapted to low soil P availability and high P fixing capacity conditions, have been suggested as a means of enhancing the solubility of less labile P forms and increasing P cycling (Almeida and Rosolem 2016), with the intention of improving P availability for subsequent cash crops. Furthermore, the use of cover crops in no-till farming system has been shown as a good strategy to reduce the soil P adsorption capacity, when compared to conventional system.

**Fig. 1:**
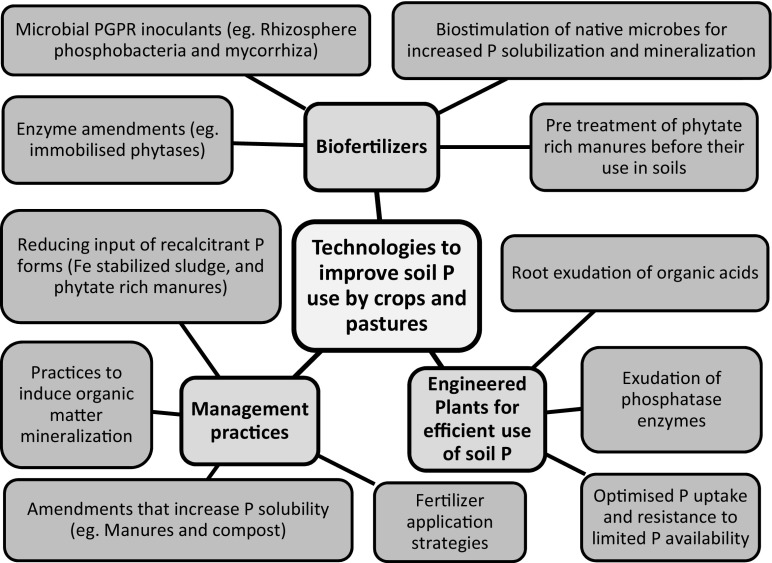
Innovations and technologies to improve soil phosphorus use by crops and pastures via: biofertilizers, engineered plants and agricultural management practices

Several different approaches are available to improve P_o_ and P_i_ availability and improve P_o_ turnover (Fig. 1). Enhancing the solubility of soil P_o_ by using amendments that alter surface properties of soil particles (Guppy et al. 2005), adding oxidizing agents, increased root exudation of organic acids, managing crop rotation and tillage, and increasing aeration and microbial respiration in soils may improve the availability of P, but may also have undesirable impacts on the carbon cycle. These include increasing organic matter loss and CO_2_ emission into the atmosphere. Little is known about whether carbon loss would be greater or less than the equivalent impact of P fertilizer application. As climate change represents another global threat for future agriculture sustainability, then innovations to improve soil P availability ideally should not induce increases in greenhouse gas emissions. Acting independently of the soils C:P stoichiometry, enzyme related technologies can release P_i_ from soil P_o_ without affecting stabilized organic carbon and therefore appear to be a favourable approach to mobilizing a significant fraction of the residual P_o_ without causing loss of carbon to the atmosphere (Trouillefou et al. 2015).

If an amendment is applied to increase soil P solubilisation, desorption rates and bioavailability (Chasse and Ohno 2016; Edwards et al. 2016; Guppy et al. 2005) it would arguably also increase P losses (Nest et al. 2014) through leaching and runoff, and therefore would possibly aggravate the diffuse nutrient pollution of receiving waters and reduce the sustainability of agriculture. Considering both fresh water and oceans, current planetary conditions exceed all boundaries for P discharges (Carpenter and Bennett 2011). Due to these environmental pressures, soil P mobilization solutions should be targeted in the rhizosphere to guarantee that most of the mobilized P is taken-up by plants (Giles et al. 2017, 2016; Stutter et al. 2012). Many plant-evolved mechanisms to cope with P deficiency have been described, including modified root architecture, abundance of root hairs, root depth distribution, and P mobilisation by root exudation of enzymes, organic acids, siderophores, surfactants and microbial growth stimulants (Brown et al. 2013; Hinsinger 2001; Richardson et al. 2009; Vance et al. 2003). There is a general assumption in the scientific literature that after continuous selection of crop lineages under P sufficient conditions, modern cultivars have become ‘lazy’ in scavenging recalcitrant soil P, meaning that P mobilizing traits have become either lost or are not sufficiently expressed in most commercial plant varieties (Menezes-Blackburn et al. 2016b). Some of these plant mechanisms can still be widely enhanced in crops either by selective breeding or by genetic modification (Richardson et al. 2009) in order to develop genotypes which can cope with reduced P inputs. Similar, and sometimes more specialized, soil P scavenging traits can be found in microbes. The genetic modification of plants to express microbial traits, such as root exudation of appropriate enzymes and organic acids, is in theory a good approach for mobilizing soil fixed P (Richardson et al. 2009). Nevertheless, in most countries genetically modified (GM) food products still encounter strong public resistance and prohibitive legislative environments, regardless of the source and benefits of the genes being modified (Frewer et al. 2004). While countries with greater acceptance of GM products can (but not necessarily will) use plants expressing more efficient microbial traits, currently most countries will have to rely only on traits evolved within the same plant species.

From a technical point of view, the genetic modification of plants to express root exudation traits favouring greater P mobilization and uptake efficiency is also not a simple challenge, and many problems may render them ineffective. These include: a) insufficient expression of the trait to translate into increased P mobilisation (Menezes-Blackburn et al. 2016b); b) uneven distribution of the trait expression in roots causing insufficient spatial coverage; c) co-expression of complementary or synergistic traits may be needed for the application to successfully work in soil environments, such as the co-expression of organic acids and phytases (Giles et al. 2017); d) the expressed trait may cause a down regulation of the rhizosphere microbial expression of the same trait (unpublished); c) unforeseen interactions of exuded biomolecules in different soil environments, such as unfavourable changes in soil pH (Giles et al. 2017), enzyme inactivation after adsorption into solid soil phase (George et al. 2006b) or immediate microbial degradation of the exuded biomolecule (Menezes-Blackburn et al. 2016b); d) unforeseen negative effects related to the function of the rhizosphere microbes, such as increased immobilization of P in the microbial biomass (Menezes-Blackburn et al. 2016b); e) the genetic modification may represent ‘too big’ an energetic/ biochemical burden to the plant, overcoming its benefits (Hu and Du 2006); f) unintended plant physiological changes are observed even in vector controls, lacking the heterologous expression of the targeted functional gene, which can cause them to underperform compared to the wild type controls in terms of P uptake (Giles et al. 2016).

In consideration of these complicated issues, the optimum approach may not be to directly mobilize soil P at all, but to reduce plant requirements for high P availability in soils. A promising biotechnological approach derives from genetic studies to develop crops with reduced phosphate accumulation in the form of phytate in grains (Raboy 2001, 2002). On the other hand, reducing phytate levels in seeds may have unintended consequences for germination and seedling vigour. However, a reduction in plant requirements for high P availability in soils would allow productivity to be maintained at a reduced P fertilizer input, depleting available P and reversing the equilibrium towards a natural and gradual mobilization of fixed soil P by crops.

Inoculating the soil with microbes screened for traits that favour the efficient mobilization of recalcitrant P has been widely proposed, and some phosphobacteria and mycorrhizal inoculants are already commercially available (Owen et al. 2015). Nevertheless, these inoculants have to compete with native soil microbes and a few important issues still keep this technology from being the decisive solution for accessing soil fixed P (Jakobsen et al. 2005), including: a) limited impact on plant growth and therefore limited commercial value; b) plant-inoculant specificity; c) inefficient colonization of rhizospheres and small inoculum survival (Martinez-Viveros et al. 2010). Even when enough P is released by the microbe inoculants, parallel P fixation in the microbial biomass can negate plant growth and P uptake (Menezes-Blackburn et al. 2014). Recent developments have demonstrated that using phosphobacteria inoculants along with their grazers (nematodes) could significantly increase available P and plant P uptake (Irshad et al. 2012). This work underlines the importance of trophic cascades to avoid/diminish competition of plant and microbial inoculants and increase the cycling of released P.

Fertilizer application technologies such as rate, frequency, depth and fertilizer placement relative to seed position have an important effect on P uptake efficiency, and are dependent on both plant and soil type. The fertilizer should be applied where and when the plants need it; applying P fertilizer to the whole topsoil is not an efficient approach and the rhizosphere should ideally be targeted. At the field scale, one way to manage the heterogeneity of P in agricultural soils is through precision farming, whereby the distribution of bioavailable P in the topsoil is accurately assessed by soil testing, and fertiliser spread at appropriate rates accordingly (Carr et al. 1991; Wollenhaupt et al. 1994).

## The need for a new understanding of the bioavailability of phosphorus pools

Not only effective biotechnologies for soil phosphorus mobilization are needed but also better management practices. In order to intervene in the fixation and soil recalcitrance of different P species there is a need for better management of P fertilizer application in arable and grassland soils. Fertilizer recommendations can vary greatly (up to 3-fold) for the same P status (Often derived by Olsen extraction) (Jordan-Meille et al. 2012), and better ways of assessing P bioavailability, linked to clear criteria for P fertilizer application rates, still do not exist after decades of related research (Beegle 2005; Six et al. 2013). The main reason is that most soil agronomic P tests tend to poorly represent the plant P uptake across different soils and only work well for limited soil and plant combinations under increasing P fertilizer doses because they are derived from limited classical critical P experiments. In the same sense, there is still not a well validated, universal soil test that represents soil P saturation and potential for P loss to receiving waters (Maguire et al. 2005). Our conceptual understanding of P cycling and bioavailability based on static pools (that can be represented by single soil test) needs to be revised and updated in order to better inform our management strategies for sustainable management of our natural resources.

**Fig. 2:**
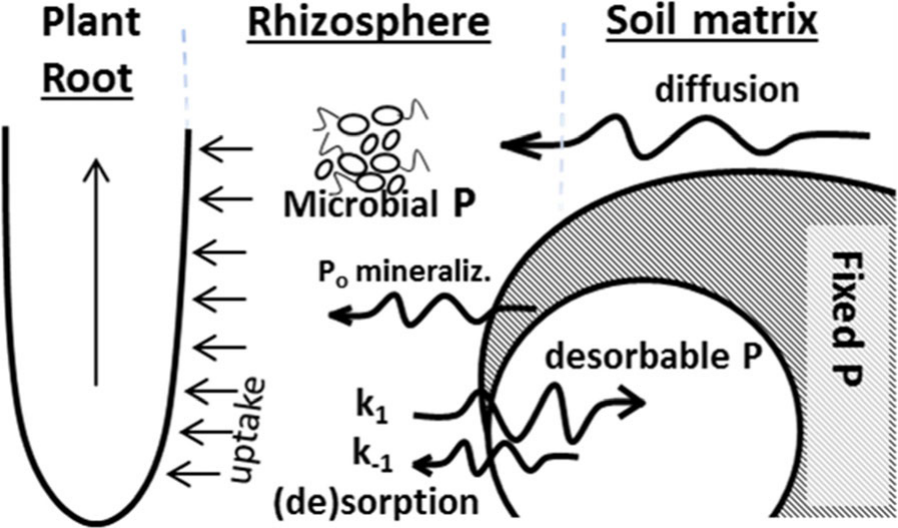
Rhizosphere processes involved in soil phosphorus bioavailability and plant uptake: diffusion through soil solution; sorption desorption balance; organic phosphorus (P_o_) mineralization; and fixation in microbial biomass

Plant roots can deplete rhizosphere solution P in a matter of minutes (Oehl et al. 2001), and therefore soil P fertility is actually not only a function of a “pool size” but of the rate at which P can move to the rhizosphere by diffusion and desorption following depletion (Kovar and Claassen 2005). Understanding the nature of P availability as an integration of kinetic rhizosphere processes (Fig. 2; rate of diffusion, desorption and mineralization) is a critical change of mind-set for the current P research community (Menezes-Blackburn et al. 2016c). Soil P pools are traditionally viewed as a range of static, largely isolated groups of P species, separated by their chemical lability. Chemical lability is defined as how likely these P forms are to undergo a change of state, such as adsorbed-to-desorbed or precipitated-to-soluble, and is normally poorly assessed by quantifying equilibrium solution P after shaking these soils with different extractants. From their chemical lability, plant bioavailability is estimated, and may be arbitrarily classified on a gradient of increasing lability and plant (crop) bioavailability pools, such as the ones described by Johnston et al. (2014): a) immediately accessible – soil solution P or water extractable P; b) large accessibility - readily available and extractable by agronomic P tests such as Olsen P; c) limited accessibility - less readily available, strongly bonded and adsorbed P; d) very limited accessibility – mostly unavailable, very strongly bound to soil solid phase, mineral P or insoluble P precipitates (Johnston et al. 2014).

This mechanistic approach of using 2 to 4 lability compartments (pools) to define plant bioavailability has been proven to be compatible with the different methods of extracting P (such as Olsen and Morgan tests), and has also proven useful to some extent in managing the fertilizer dose needed to sustain adequate crop productivity (Johnston et al. 2014; Syers et al. 2008). Nevertheless, when it comes to understanding the system dynamics and the accumulation / mobilisation of soil P over years, this approach is simplistic and limits the current understanding in several ways: a) it mostly ignores the role of organic phosphorus and P forms locked in the soil microbial biomass; b) the chemical speciation of P_i_ and P_o_ and their different behaviour is only dealt in a very superficial way (e.g. acid vs alkali solubility and different extractant strength); c) when dealing with readily bioavailable pools, this model ignores the abundance of the different chemical P species and their kinetics of diffusion, desorption and solubilisation; d) information about the effect of size of chemical P complexes and their aggregates on P lability is overlooked; e) this type of model only deals with complexity by increasing the number of lability compartments, which does not directly represent soil processes or their integration (solubilisation, diffusion, desorption, mineralization, uptake, etc.); f) it ignores plant mechanisms to actively mobilize P through root conditioning of the rhizosphere environment such as pH change, exudation of organic acids and enzymes (Darch et al. 2016).

An improved conceptual model of P cycling in soils is needed in order to improve our understanding of soil P accumulation and to address the limited knowledge on soil P bioavailability by improving fertilizer P use efficiency. In this new conceptual model of P cycling, a temporal (kinetic) component of soil P transformations must be considered. Zheng and Zhang (2011) made an attempt to associate the Hedley sequential fractionation with chemical lability pools, categorized in *slow* and *rapid* cycling depending on the strength of the extractant (Hedley et al. 1982; Zheng and Zhang 2011). This type of pool fractionation analysis is misleading, does not capture the real processes occurring in soils and often leads to speculative discussions about their bioavailability and chemical lability (Turner et al. 2005). On the other hand, giving a kinetic component to this analysis is an improvement on the static lability pools, and there is still much to be uncovered about the behaviour of individual chemical P species. For an accurate and correct interpretation of the system, coupled with better assessment of bioavailability, a complete speciation of soil P must be made with the characterization of the temporal dynamics of individual soil P species and their rates of cycling. This will allow the assessment of possible interventions on the soil P cycle to alter input/output balances of rapid cycling species (quasi instant and daily) and or intervene on accumulation/depletion of slower cycling pools (seasonal and inter-annual).

## Future perspectives for the biotechnological mobilization of soil phosphorus

Soil P research up to the 1970s was driven by the question of how much fertilizer P was needed in order to secure maximum crop productivity. A second wave of research was driven by environmental concerns about the high P status of many fertilized soils and the resulting nutrient pollution of receiving watercourses. Both are still valid scientific questions: there is a strong need to reduce total P in soils to environmentally acceptable levels, whilst maintaining optimal crop growth conditions (Barberis et al. 1995). Nevertheless, the time has come to move on from simply understanding the behaviour, movement and transport of P in soil systems to taking action by developing technologies to enhance the efficiency of P fertilizer application and the use of our natural rock phosphate resources. In many ways, the scientific community is starting to address this demand. Nonetheless, satisfactory solutions/technologies have not yet been developed and breakthroughs are still needed. Many meetings and symposia have been held recently on the subject of soil P resulting in an increase in international cooperation on this topic. However much of the ongoing research is still fragmented and disconnected. Independently of the approach taken, researchers have a natural tendency to process information at increasingly finer scales, focusing on their individual sub-disciplines (e.g. microbial ecology, enzymology, chemical speciation, method development, etc.). Additionally, it is our appreciation that researchers in general are moved by their curiosity rather than by their willingness to generate impact. The final application or purpose of the knowledge generated tends therefore to become more often an “introduction material” rather than the actual focus of the research. In other words, new ‘big picture’ driven and impact focused research is needed if we are to create solutions for the sustainable use of the legacy soil P.

Although there is clear evidence that long term fertilizer application leads to soil P accumulation, the size and potential uses of residual soil P pools worldwide are still unknown. The analysis presented in Table 1 indicates the huge potential for using soil residual P, but the limitations inherent in this analysis mean it is insufficient for making an accurate assessment of the actual size and distribution of the legacy P pool. There is a need for building a world soil P inventory considering plant P availability indices, speciation of P forms and more importantly the size of the residual P pool that can potentially be mobilized by different technologies. Optimistically, we expect that wider multidisciplinary initiatives will soon be funded and important steps can be taken in the direction of a positive outcome on soil P mobilization technologies.

## Electronic supplementary material

ESM 1 (DOCX 205 kb)
