# Supplementary material for: Opportunities for mobilizing recalcitrant phosphorus from agricultural soils: a review
Source: Plant Soil. 2017 Aug 1;427(1):5–16. doi: 10.1007/s11104-017-3362-2 (PMC6438637; doi:10.1007/s11104-017-3362-2)
Supplement: Supplementary file 1 — (DOCX 205 kb) [file 11104_2017_3362_MOESM1_ESM.docx]

**SUPPLEMENTARY MATERIAL**

**Table S1.** Literature data compilation of phosphorus stocks analysed by ^31^P-NMR from arable and pasture soils.

| **Continent** | **Country** | **Land Use** | **Total P** | **Inorganic orthophosphate P** | **P Monoesters** | **P Diesters** | **Total P** | | **Inorganic orthophosphate P** | **P Monoesters** | **P Diesters** | **Other P** | **Reference** |  |  |
| --- | --- | --- | --- | --- | --- | --- | --- | --- | --- | --- | --- | --- | --- | --- | --- |
|  |  |  |  |  |  |  |  |  |  |  |  |  |  |  |  |
|  |  |  | **mg P /kg** | **------------%-----------** | | | | **-----------------Stocks kg/ha in 15cm-------------** | | | | |  | |  |
| **North America** | Canada | Crop | 171 | 42 | 58 | 0 | | 319 | 134 | 186 | 0 | 0 | (Condron et al. 1990) | | |
| **North America** | Canada | Crop | 224 | 68 | 32 | 0 | | 382 | 259 | 123 | 0 | 0 | (Condron et al. 1990) | | |
| **North America** | Canada | Crop | 146 | 50 | 50 | 0 | | 263 | 131 | 131 | 0 | 0 | (Condron et al. 1990) | | |
| **North America** | Canada | Crop | 980 | 72 | 25 | 2 | | 1762 | 1269 | 440 | 35 | 18 | (Cade-Menun et al. 2010) | | |
| **North America** | USA | Crop | 568 | 90 | 10 | 0 | | 1293 | 1164 | 129 | 0 | 0 | (Turner et al. 2003a) | | |
| **North America** | USA | Crop | 220 | 70 | 26 | 0 | | 490 | 343 | 127 | 0 | 20 | (Turner et al. 2003a) | | |
| **North America** | USA | Crop | 827 | 66 | 31 | 2 | | 1780 | 1175 | 552 | 36 | 18 | (Turner et al. 2003a) | | |
| **North America** | USA | Crop | 1210 | 76 | 23 | 0 | | 2605 | 1980 | 599 | 0 | 26 | (Turner et al. 2003a) | | |
| **North America** | USA | Crop | 251 | 74 | 26 | 0 | | 540 | 400 | 140 | 0 | 0 | (Turner et al. 2003a) | | |
| **North America** | USA | Crop | 970 | 77 | 22 | 0 | | 2088 | 1608 | 459 | 0 | 21 | (Turner et al. 2003a) | | |
| **North America** | USA | Crop | 1058 | 81 | 17 | 2 | | 2244 | 1817 | 381 | 45 | 0 | (Turner et al. 2003a) | | |
| **North America** | USA | Crop | 439 | 56 | 41 | 1 | | 906 | 507 | 371 | 9 | 18 | (Turner et al. 2003a) | | |
| **North America** | USA | Crop | 1135 | 83 | 15 | 0 | | 2342 | 1944 | 351 | 0 | 47 | (Turner et al. 2003a) | | |
| **North America** | USA | Crop | 966 | 77 | 22 | 0 | | 1993 | 1535 | 439 | 0 | 20 | (Turner et al. 2003a) | | |
| **North America** | USA | Crop | 729 | 74 | 25 | 0 | | 1485 | 1099 | 371 | 0 | 15 | (Turner et al. 2003a) | | |
| **North America** | USA | Crop | 1070 | 77 | 23 | 0 | | 2180 | 1679 | 501 | 0 | 0 | (Turner et al. 2003a) | | |
| **North America** | USA | Crop | 762 | 73 | 27 | 0 | | 1534 | 1120 | 414 | 0 | 0 | (Turner et al. 2003a) | | |
| **North America** | USA | Crop | 890 | 77 | 22 | 0 | | 1732 | 1334 | 381 | 0 | 17 | (Turner et al. 2003a) | | |
| **North America** | USA | Crop | 626 | 69 | 30 | 1 | | 1218 | 841 | 365 | 12 | 0 | (Turner et al. 2003a) | | |
| **North America** | USA | Crop | 1000 | 58 | 38 | 1 | | 1850 | 1073 | 703 | 19 | 56 | (Turner et al. 2003a) | | |
| **North America** | USA | Crop | 1000 | 54 | 40 | 1 | | 1736 | 937 | 694 | 17 | 87 | (Turner et al. 2003a) | | |
| **North America** | USA | Crop | 657 | 49 | 44 | 3 | | 1084 | 531 | 477 | 33 | 43 | (Turner et al. 2003a) | | |
| **North America** | Canada | Crop | 1150 | 38 | 60 | 2 | | 2048 | 780 | 1229 | 39 | 0 | (Abdi et al. 2014) | | |
| **North America** | Canada | Crop | 547 | 46 | 49 | 4 | | 984 | 455 | 482 | 37 | 10 | (Liu et al. 2014) | | |
| **North America** | USA | Crop | 538 | 69 | 24 | 2 | | 1031 | 711 | 247 | 21 | 52 | (Hill and Cade-Menun 2009) | | |
| **North America** | USA | Crop | 1094 | 82 | 11 | 2 | | 1990 | 1632 | 219 | 40 | 99 | (Hill and Cade-Menun 2009) | | |
| **North America** | USA | Crop | 1622 | 14 | 45 | 14 | | 2097 | 289 | 946 | 287 | 575 | (Lehmann et al. 2005) | | |
| **North America** | USA | Crop | 2087 | 12 | 48 | 13 | | 2416 | 297 | 1148 | 317 | 655 | (Lehmann et al. 2005) | | |
| **North America** | USA | Crop | 3056 | 13 | 48 | 12 | | 3951 | 498 | 1904 | 478 | 1071 | (Lehmann et al. 2005) | | |
| **North America** | USA | Crop | 3087 | 18 | 52 | 11 | | 3791 | 663 | 1967 | 398 | 762 | (Lehmann et al. 2005) | | |
| **North America** | USA | Crop | 4451 | 17 | 54 | 9 | | 6014 | 1010 | 3242 | 535 | 1227 | (Lehmann et al. 2005) | | |
| **North America** | USA | Crop | 6064 | 15 | 51 | 14 | | 7178 | 1069 | 3661 | 983 | 1464 | (Lehmann et al. 2005) | | |
| **North America** | USA | Crop | 7802 | 20 | 48 | 9 | | 9794 | 1949 | 4721 | 921 | 2204 | (Lehmann et al. 2005) | | |
| **Europe** | Germany | Crop | 1220 | 16 | 52 | 21 | | 2136 | 342 | 1111 | 449 | 235 | (Guggenberger et al. 1996a) | | |
| **Europe** | DK | Crop | 511 | 26 | 47 | 9 | | 994 | 259 | 467 | 90 | 179 | (Guggenberger et al. 1996a) | | |
| **Europe** | DK | Crop | 786 | 31 | 44 | 9 | | 1530 | 474 | 673 | 138 | 245 | (Guggenberger et al. 1996a) | | |
| **Europe** | DK | Crop | 783 | 49 | 35 | 6 | | 1477 | 724 | 517 | 89 | 148 | (Guggenberger et al. 1996a) | | |
| **Europe** | UK | Crop | 3435 | 85 | 14 | 1 | | 5962 | 5068 | 859 | 36 | 0 | (Stutter et al. 2015) | | |
| **Europe** | UK | Crop | 1775 | 64 | 35 | 1 | | 3193 | 2031 | 1123 | 40 | 0 | (Stutter et al. 2015) | | |
| **Europe** | UK | Crop | 1492 | 65 | 35 | 0 | | 2568 | 1672 | 896 | 0 | 0 | (Stutter et al. 2015) | | |
| **Europe** | UK | Crop | 1486 | 91 | 9 | 0 | | 2832 | 2568 | 264 | 0 | 0 | (Stutter et al. 2015) | | |
| **Europe** | UK | Crop | 1475 | 76 | 24 | 0 | | 2678 | 2044 | 634 | 0 | 0 | (Stutter et al. 2015) | | |
| **Europe** | UK | Crop | 1193 | 66 | 32 | 1 | | 2207 | 1453 | 709 | 31 | 15 | (Stutter et al. 2015) | | |
| **Europe** | UK | Crop | 1078 | 69 | 31 | 0 | | 2121 | 1474 | 647 | 0 | 0 | (Stutter et al. 2015) | | |
| **Europe** | UK | Crop | 898 | 68 | 32 | 0 | | 1586 | 1075 | 512 | 0 | 0 | (Stutter et al. 2015) | | |
| **Europe** | UK | Crop | 886 | 58 | 42 | 0 | | 1538 | 887 | 650 | 0 | 0 | (Stutter et al. 2015) | | |
| **Europe** | UK | Crop | 856 | 72 | 27 | 1 | | 1448 | 1046 | 387 | 15 | 0 | (Stutter et al. 2015) | | |
| **Europe** | UK | Crop | 802 | 74 | 26 | 0 | | 1281 | 948 | 333 | 0 | 0 | (Stutter et al. 2015) | | |
| **Europe** | UK | Crop | 670 | 70 | 30 | 0 | | 1252 | 874 | 377 | 0 | 0 | (Stutter et al. 2015) | | |
| **Europe** | UK | Crop | 650 | 64 | 35 | 0 | | 1119 | 711 | 387 | 0 | 21 | (Stutter et al. 2015) | | |
| **Europe** | Switzerland | Crop | 690 | 68 | 27 | 3 | | 600 | 408 | 162 | 18 | 12 | (Annaheim et al. 2015) | | |
| **Europe** | Switzerland | Crop | 210 | 80 | 19 | 0 | | 183 | 146 | 35 | 0 | 2 | (Annaheim et al. 2015) | | |
| **Europe** | Switzerland | Crop | 3320 | 91 | 9 | 0 | | 2888 | 2628 | 260 | 0 | 0 | (Annaheim et al. 2015) | | |
| **Europe** | Sweeden | Crop | 711 | 72 | 27 | 0 | | 1256 | 910 | 334 | 0 | 12 | (Ahlgren et al. 2013) | | |
| **Europe** | Sweeden | Crop | 756 | 78 | 21 | 0 | | 1504 | 1171 | 323 | 0 | 10 | (Ahlgren et al. 2013) | | |
| **Europe** | Sweeden | Crop | 980 | 79 | 20 | 0 | | 1868 | 1468 | 378 | 0 | 22 | (Ahlgren et al. 2013) | | |
| **Europe** | Sweeden | Crop | 883 | 65 | 33 | 0 | | 1618 | 1054 | 534 | 0 | 30 | (Ahlgren et al. 2013) | | |
| **Europe** | UK | Crop | 435 | 75 | 24 | 3 | | 839 | 629 | 201 | 21 | 0 | (Bunemann et al. 2008) | | |
| **Europe** | UK | Crop | 427 | 78 | 22 | 2 | | 839 | 655 | 185 | 20 | 0 | (Bunemann et al. 2008) | | |
| **Europe** | UK | Crop | 479 | 79 | 19 | 4 | | 948 | 749 | 180 | 38 | 0 | (Bunemann et al. 2008) | | |
| **Europe** | UK | Crop | 546 | 69 | 28 | 7 | | 917 | 633 | 257 | 63 | 0 | (Bunemann et al. 2008) | | |
| **Europe** | UK | Crop | 447 | 72 | 26 | 5 | | 835 | 601 | 217 | 39 | 0 | (Bunemann et al. 2008) | | |
| **Europe** | Germany | Crop | 1863 | 81 | 15 | 4 | | 3321 | 2690 | 498 | 133 | 0 | (Leinweber et al. 1997) | | |
| **Europe** | Germany | Crop | 1469 | 81 | 16 | 2 | | 2800 | 2268 | 448 | 56 | 28 | (Leinweber et al. 1997) | | |
| **Europe** | Germany | Crop | 2249 | 77 | 14 | 1 | | 4640 | 3573 | 650 | 46 | 371 | (Leinweber et al. 1997) | | |
| **Europe** | Germany | Crop | 747 | 88 | 10 | 2 | | 1395 | 1228 | 140 | 28 | 0 | (Leinweber et al. 1997) | | |
| **Europe** | Finland | Crop | 930 | 30 | 49 | 2 | | 1385 | 416 | 679 | 28 | 263 | (Soinne et al. 2011) | | |
| **Europe** | Finland | Crop | 890 | 33 | 50 | 2 | | 1411 | 466 | 705 | 21 | 219 | (Soinne et al. 2011) | | |
| **South America** | Brazil | Crop | 321 | 44 | 37 | 10 | | 562 | 245 | 210 | 57 | 51 | (Chapuis-Lardy et al. 2001) | | |
| **South America** | Brazil | Crop | 377 | 67 | 22 | 4 | | 654 | 440 | 144 | 28 | 43 | (Chapuis-Lardy et al. 2001) | | |
| **South America** | Brazil | Crop | 346 | 47 | 35 | 10 | | 571 | 267 | 199 | 55 | 50 | (Chapuis-Lardy et al. 2001) | | |
| **South America** | Brazil | Crop | 853 | 55 | 35 | 6 | | 496 | 273 | 176 | 32 | 16 | (Gatiboni et al. 2007) | | |
| **South America** | Brazil | Crop | 915 | 59 | 32 | 5 | | 447 | 266 | 144 | 21 | 17 | (Gatiboni et al. 2007) | | |
| **South America** | Brazil | Crop | 939 | 70 | 24 | 4 | | 443 | 312 | 104 | 17 | 10 | (Gatiboni et al. 2007) | | |
| **Asia** | Thailand | Crop | 2050 | 12 | 65 | 15 | | 3030 | 364 | 1970 | 455 | 242 | (Moller et al. 2000) | | |
| **Asia** | China | Crop | 1590 | 82 | 17 | 1 | | 2760 | 2266 | 471 | 22 | 0 | (Jin et al. 2016) | | |
| **Asia** | China | Crop | 1080 | 84 | 16 | 0 | | 2093 | 1761 | 332 | 0 | 0 | (Jin et al. 2016) | | |
| **Oceania** | Australia | Crop | 506 | 64 | 19 | 0 | | 971 | 623 | 185 | 0 | 163 | (McLaren et al. 2014) | | |
| **Oceania** | Australia | Crop | 830 | 68 | 15 | 0 | | 1569 | 1064 | 234 | 0 | 271 | (McLaren et al. 2014) | | |
| **Oceania** | Australia | Crop | 1158 | 68 | 18 | 0 | | 2224 | 1508 | 405 | 0 | 311 | (McLaren et al. 2014) | | |
| **Oceania** | Australia | Crop | 697 | 60 | 36 | 0 | | 1334 | 806 | 480 | 0 | 48 | (McLaren et al. 2014) | | |
| **Oceania** | Australia | Crop | 325 | 59 | 32 | 0 | | 648 | 385 | 208 | 0 | 54 | (McLaren et al. 2014) | | |
| **Oceania** | Australia | Crop | 185 | 57 | 43 | 0 | | 371 | 211 | 160 | 0 | 0 | (McLaren et al. 2014) | | |
| **Oceania** | Australia | Crop | 388 | 73 | 22 | 5 | | 755 | 551 | 164 | 41 | 0 | (McLaren et al. 2014) | | |
| **Oceania** | Australia | Crop | 760 | 53 | 37 | 0 | | 1221 | 652 | 455 | 0 | 114 | (McLaren et al. 2014) | | |
| **Oceania** | Australia | Crop | 2012 | 74 | 13 | 0 | | 3920 | 2907 | 524 | 0 | 488 | (McLaren et al. 2014) | | |
| **Oceania** | Australia | Crop | 477 | 65 | 21 | 0 | | 940 | 613 | 193 | 0 | 133 | (McLaren et al. 2014) | | |
| **Oceania** | Australia | Crop | 1127 | 60 | 25 | 0 | | 2311 | 1390 | 582 | 0 | 338 | (McLaren et al. 2014) | | |
| **Oceania** | Australia | Crop | 271 | 70 | 25 | 3 | | 511 | 360 | 128 | 17 | 6 | (Doolette et al. 2009) | | |
| **Oceania** | Australia | Crop | 629 | 89 | 9 | 2 | | 1252 | 1109 | 111 | 19 | 13 | (Doolette et al. 2009) | | |
| **Oceania** | Australia | Crop | 622 | 88 | 10 | 1 | | 1252 | 1106 | 121 | 15 | 10 | (Doolette et al. 2009) | | |
| **Oceania** | Australia | Crop | 239 | 63 | 32 | 1 | | 451 | 284 | 144 | 5 | 18 | (Doolette et al. 2011) | | |
| **Oceania** | Australia | Crop | 743 | 85 | 14 | 1 | | 1478 | 1257 | 207 | 15 | 0 | (Doolette et al. 2011) | | |
| **Oceania** | Australia | Crop | 702 | 81 | 18 | 1 | | 1413 | 1145 | 254 | 14 | 0 | (Doolette et al. 2011) | | |
| **Oceania** | Australia | Crop | 844 | 58 | 0 | 0 | | 1547 | 897 | 0 | 0 | 650 | (Doolette et al. 2011) | | |
| **Oceania** | Australia | Crop | 571 | 74 | 0 | 0 | | 1067 | 789 | 0 | 0 | 277 | (Doolette et al. 2011) | | |
| **Oceania** | NZ | Crop | 857 | 53 | 44 | 0 | | 1193 | 632 | 525 | 0 | 36 | (McDowell and Koopmans 2006) | | |
| **Africa** | Tanzania | Crop | 489 | 53 | 41 | 6 | | 1009 | 535 | 414 | 61 | 0 | (Solomon and Lehman 2000) | | |
| **Africa** | Tanzania | Crop | 445 | 55 | 39 | 6 | | 918 | 505 | 358 | 55 | 0 | (Solomon and Lehman 2000) | | |
| **Africa** | Ethiopa | Crop | 927 | 9 | 61 | 17 | | 1446 | 130 | 882 | 246 | 188 | (Solomon et al. 2002) | | |
| **Africa** | Ethiopa | Crop | 874 | 8 | 66 | 16 | | 1364 | 109 | 900 | 218 | 136 | (Solomon et al. 2002) | | |
| **Africa** | Kenia | Crop | 757 | 33 | 56 | 4 | | 790 | 261 | 443 | 35 | 51 | (George et al. 2006) | | |
| **Africa** | Madagascar | Crop | 337 | 70 | 30 | 0 | | 670 | 469 | 201 | 0 | 0 | (Turner 2006) | | |
| **Africa** | Madagascar | Crop | 516 | 67 | 31 | 0 | | 967 | 648 | 300 | 0 | 19 | (Turner 2006) | | |
| **Africa** | Madagascar | Crop | 417 | 65 | 30 | 4 | | 754 | 490 | 226 | 30 | 8 | (Turner 2006) | | |
| **Africa** | Madagascar | Crop | 531 | 80 | 16 | 2 | | 1014 | 811 | 162 | 20 | 20 | (Turner 2006) | | |
| **Africa** | Madagascar | Crop | 697 | 72 | 24 | 0 | | 1287 | 927 | 309 | 0 | 51 | (Turner 2006) | | |
| **Africa** | Madagascar | Crop | 305 | 61 | 28 | 8 | | 537 | 328 | 150 | 43 | 16 | (Turner 2006) | | |
| **Africa** | Madagascar | Crop | 316 | 54 | 38 | 6 | | 560 | 303 | 213 | 34 | 11 | (Turner 2006) | | |
| **Africa** | Madagascar | Crop | 828 | 70 | 24 | 5 | | 1060 | 742 | 254 | 53 | 11 | (Turner 2006) | | |
| **Africa** | Madagascar | Crop | 1378 | 64 | 28 | 7 | | 859 | 550 | 241 | 60 | 9 | (Turner 2006) | | |
| **Africa** | Madagascar | Crop | 1128 | 77 | 19 | 3 | | 1825 | 1406 | 347 | 55 | 18 | (Turner 2006) | | |
| **Africa** | Madagascar | Crop | 189 | 56 | 31 | 9 | | 341 | 191 | 106 | 31 | 14 | (Turner 2006) | | |
| **Africa** | Madagascar | Crop | 133 | 74 | 24 | 0 | | 264 | 196 | 63 | 0 | 5 | (Turner 2006) | | |
| **Africa** | Madagascar | Crop | 188 | 66 | 25 | 4 | | 352 | 232 | 88 | 14 | 18 | (Turner 2006) | | |
| **Europe** | Germany | Pasture | 2063 | 15 | 55 | 22 | | 2336 | 350 | 1285 | 514 | 187 | (Guggenberger et al. 1996a) | | |
| **Europe** | Germany | Pasture | 4981 | 82 | 16 | 3 | | 7895 | 6474 | 1263 | 237 | 0 | (Leinweber et al. 1997) | | |
| **Europe** | Germany | Pasture | 965 | 44 | 42 | 15 | | 1592 | 701 | 669 | 239 | 0 | (Leinweber et al. 1997) | | |
| **Europe** | UK | Pasture | 1020 | 40 | 51 | 2 | | 1713 | 685 | 873 | 34 | 120 | (Turner et al. 2003b) | | |
| **Europe** | UK | Pasture | 519 | 33 | 50 | 6 | | 866 | 286 | 433 | 52 | 95 | (Turner et al. 2003b) | | |
| **Europe** | UK | Pasture | 834 | 55 | 34 | 4 | | 1381 | 759 | 469 | 55 | 97 | (Turner et al. 2003b) | | |
| **Europe** | UK | Pasture | 1021 | 39 | 48 | 4 | | 1688 | 658 | 810 | 68 | 152 | (Turner et al. 2003b) | | |
| **Europe** | UK | Pasture | 923 | 46 | 40 | 8 | | 1501 | 690 | 600 | 120 | 90 | (Turner et al. 2003b) | | |
| **Europe** | UK | Pasture | 585 | 30 | 60 | 3 | | 921 | 276 | 552 | 28 | 64 | (Turner et al. 2003b) | | |
| **Europe** | UK | Pasture | 821 | 55 | 29 | 6 | | 1272 | 700 | 369 | 76 | 127 | (Turner et al. 2003b) | | |
| **Europe** | UK | Pasture | 376 | 31 | 58 | 4 | | 578 | 179 | 335 | 23 | 40 | (Turner et al. 2003b) | | |
| **Europe** | UK | Pasture | 568 | 36 | 50 | 3 | | 872 | 314 | 436 | 26 | 96 | (Turner et al. 2003b) | | |
| **Europe** | UK | Pasture | 821 | 27 | 60 | 4 | | 1434 | 387 | 860 | 57 | 129 | (Turner et al. 2003b) | | |
| **Europe** | UK | Pasture | 988 | 44 | 46 | 4 | | 1475 | 649 | 678 | 59 | 88 | (Turner et al. 2003b) | | |
| **Europe** | UK | Pasture | 962 | 38 | 49 | 5 | | 1433 | 544 | 702 | 72 | 115 | (Turner et al. 2003b) | | |
| **Europe** | UK | Pasture | 1321 | 50 | 41 | 5 | | 1954 | 977 | 801 | 98 | 78 | (Turner et al. 2003b) | | |
| **Europe** | UK | Pasture | 571 | 23 | 60 | 7 | | 841 | 194 | 505 | 59 | 84 | (Turner et al. 2003b) | | |
| **Europe** | UK | Pasture | 1106 | 39 | 48 | 7 | | 1622 | 633 | 779 | 114 | 97 | (Turner et al. 2003b) | | |
| **Europe** | UK | Pasture | 1312 | 52 | 39 | 4 | | 1907 | 992 | 744 | 76 | 95 | (Turner et al. 2003b) | | |
| **Europe** | UK | Pasture | 854 | 23 | 59 | 10 | | 1241 | 286 | 732 | 124 | 99 | (Turner et al. 2003b) | | |
| **Europe** | UK | Pasture | 887 | 31 | 55 | 7 | | 1287 | 399 | 708 | 90 | 90 | (Turner et al. 2003b) | | |
| **Europe** | UK | Pasture | 833 | 41 | 48 | 4 | | 1204 | 493 | 578 | 48 | 84 | (Turner et al. 2003b) | | |
| **Europe** | UK | Pasture | 997 | 38 | 48 | 6 | | 1436 | 546 | 689 | 86 | 115 | (Turner et al. 2003b) | | |
| **Europe** | UK | Pasture | 626 | 39 | 53 | 3 | | 894 | 349 | 474 | 27 | 45 | (Turner et al. 2003b) | | |
| **Europe** | UK | Pasture | 1004 | 28 | 54 | 7 | | 1367 | 383 | 738 | 96 | 150 | (Turner et al. 2003b) | | |
| **Europe** | UK | Pasture | 1524 | 26 | 60 | 7 | | 2034 | 529 | 1220 | 142 | 142 | (Turner et al. 2003b) | | |
| **Europe** | UK | Pasture | 1074 | 36 | 53 | 6 | | 1417 | 510 | 751 | 85 | 71 | (Turner et al. 2003b) | | |
| **Europe** | UK | Pasture | 784 | 21 | 58 | 9 | | 1003 | 211 | 582 | 90 | 120 | (Turner et al. 2003b) | | |
| **Europe** | UK | Pasture | 900 | 32 | 48 | 9 | | 1136 | 363 | 545 | 102 | 125 | (Turner et al. 2003b) | | |
| **Europe** | UK | Pasture | 989 | 31 | 55 | 6 | | 1235 | 383 | 679 | 74 | 99 | (Turner et al. 2003b) | | |
| **Europe** | UK | Pasture | 1007 | 23 | 59 | 7 | | 1248 | 287 | 736 | 87 | 137 | (Turner et al. 2003b) | | |
| **Europe** | UK | Pasture | 1981 | 36 | 48 | 7 | | 2253 | 811 | 1082 | 158 | 203 | (Turner et al. 2003b) | | |
| **Europe** | UK | Pasture | 1865 | 55 | 41 | 2 | | 2635 | 1458 | 1084 | 55 | 38 | (Stutter et al. 2015) | | |
| **Europe** | UK | Pasture | 1341 | 39 | 54 | 3 | | 2109 | 830 | 1132 | 74 | 74 | (Stutter et al. 2015) | | |
| **Europe** | UK | Pasture | 1327 | 62 | 36 | 0 | | 1293 | 796 | 461 | 0 | 35 | (Stutter et al. 2015) | | |
| **Europe** | UK | Pasture | 1253 | 26 | 58 | 7 | | 1527 | 403 | 886 | 104 | 135 | (Stutter et al. 2015) | | |
| **Europe** | UK | Pasture | 1233 | 58 | 38 | 0 | | 1536 | 898 | 577 | 0 | 62 | (Stutter et al. 2015) | | |
| **Europe** | UK | Pasture | 1169 | 48 | 48 | 2 | | 1689 | 803 | 816 | 26 | 45 | (Stutter et al. 2015) | | |
| **Europe** | UK | Pasture | 977 | 53 | 43 | 0 | | 1433 | 762 | 619 | 0 | 52 | (Stutter et al. 2015) | | |
| **Europe** | UK | Pasture | 901 | 36 | 58 | 3 | | 1621 | 584 | 932 | 45 | 60 | (Stutter et al. 2015) | | |
| **Europe** | UK | Pasture | 898 | 71 | 29 | 0 | | 1317 | 936 | 379 | 0 | 2 | (Stutter et al. 2015) | | |
| **Europe** | UK | Pasture | 886 | 64 | 34 | 0 | | 1579 | 1017 | 531 | 0 | 32 | (Stutter et al. 2015) | | |
| **Europe** | UK | Pasture | 1431 | 31 | 58 | 8 | | 1245 | 391 | 727 | 95 | 32 | (Stutter et al. 2015) | | |
| **Europe** | UK | Pasture | 1416 | 28 | 54 | 8 | | 1245 | 350 | 673 | 96 | 127 | (Stutter et al. 2015) | | |
| **Europe** | UK | Pasture | 1324 | 24 | 43 | 12 | | 1590 | 376 | 681 | 187 | 345 | (Stutter et al. 2015) | | |
| **Europe** | UK | Pasture | 1238 | 22 | 56 | 9 | | 160 | 35 | 89 | 15 | 20 | (Stutter et al. 2015) | | |
| **Europe** | UK | Pasture | 643 | 33 | 56 | 6 | | 943 | 309 | 531 | 53 | 51 | (Stutter et al. 2015) | | |
| **Europe** | UK | Pasture | 294 | 64 | 36 | 0 | | 529 | 341 | 188 | 0 | 0 | (Stutter et al. 2015) | | |
| **Europe** | Ireland | Pasture | 819 | 49 | 43 | 4 | | 1340 | 654 | 582 | 59 | 46 | (Bourke et al. 2008) | | |
| **Europe** | Netherlands | Pasture | 393 | 48 | 52 | 0 | | 543 | 262 | 281 | 0 | 0 | (Koopmans et al. 2003) | | |
| **Europe** | Netherlands | Pasture | 436 | 46 | 52 | 0 | | 580 | 266 | 304 | 0 | 10 | (Koopmans et al. 2003) | | |
| **Europe** | Netherlands | Pasture | 349 | 34 | 61 | 0 | | 435 | 150 | 264 | 0 | 21 | (Koopmans et al. 2003) | | |
| **Europe** | Netherlands | Pasture | 742 | 71 | 29 | 0 | | 959 | 684 | 275 | 0 | 0 | (Koopmans et al. 2003) | | |
| **Europe** | Netherlands | Pasture | 2400 | 83 | 16 | 0 | | 3057 | 2541 | 477 | 6 | 34 | (Koopmans et al. 2003) | | |
| **Europe** | Netherlands | Pasture | 2007 | 77 | 18 | 0 | | 2501 | 1926 | 448 | 0 | 128 | (Koopmans et al. 2003) | | |
| **Europe** | Netherlands | Pasture | 1658 | 82 | 14 | 0 | | 2191 | 1788 | 296 | 0 | 107 | (Koopmans et al. 2003) | | |
| **Europe** | Netherlands | Pasture | 1134 | 76 | 19 | 3 | | 1532 | 1163 | 294 | 49 | 26 | (Koopmans et al. 2003) | | |
| **Europe** | Netherlands | Pasture | 1222 | 66 | 27 | 0 | | 1534 | 1017 | 408 | 0 | 109 | (Koopmans et al. 2003) | | |
| **Europe** | Ireland | Pasture | 616 | 40 | 52 | 0 | | 1042 | 417 | 542 | 0 | 83 | (Murphy et al. 2009) | | |
| **Europe** | Ireland | Pasture | 1638 | 57 | 41 | 0 | | 2440 | 1391 | 1000 | 0 | 49 | (Murphy et al. 2009) | | |
| **Europe** | Ireland | Pasture | 1488 | 47 | 49 | 2 | | 2216 | 1042 | 1086 | 44 | 44 | (Murphy et al. 2009) | | |
| **Europe** | Ireland | Pasture | 2580 | 78 | 22 | 0 | | 3360 | 2621 | 739 | 0 | 0 | (Murphy et al. 2009) | | |
| **Europe** | Ireland | Pasture | 1159 | 86 | 14 | 0 | | 2256 | 1940 | 316 | 0 | 0 | (Murphy et al. 2009) | | |
| **Europe** | Ireland | Pasture | 779 | 34 | 64 | 0 | | 1329 | 452 | 851 | 0 | 27 | (Murphy et al. 2009) | | |
| **Europe** | Ireland | Pasture | 844 | 31 | 59 | 3 | | 1881 | 583 | 1110 | 56 | 132 | (Murphy et al. 2009) | | |
| **Europe** | Ireland | Pasture | 1330 | 50 | 42 | 5 | | 2027 | 1014 | 851 | 101 | 61 | (Murphy et al. 2009) | | |
| **Europe** | Ireland | Pasture | 1067 | 26 | 71 | 2 | | 1601 | 416 | 1137 | 32 | 16 | (Murphy et al. 2009) | | |
| **Europe** | Ireland | Pasture | 1088 | 37 | 58 | 3 | | 1621 | 600 | 940 | 49 | 32 | (Murphy et al. 2009) | | |
| **Europe** | Ireland | Pasture | 1362 | 48 | 47 | 1 | | 2029 | 974 | 953 | 20 | 81 | (Murphy et al. 2009) | | |
| **Europe** | Ireland | Pasture | 1472 | 53 | 42 | 1 | | 2192 | 1162 | 921 | 22 | 88 | (Murphy et al. 2009) | | |
| **Europe** | Ireland | Pasture | 1298 | 37 | 51 | 6 | | 1876 | 694 | 957 | 113 | 113 | (Murphy et al. 2009) | | |
| **Europe** | Ireland | Pasture | 1508 | 47 | 48 | 3 | | 2115 | 994 | 1015 | 63 | 42 | (Murphy et al. 2009) | | |
| **Europe** | Ireland | Pasture | 1458 | 35 | 62 | 0 | | 1985 | 695 | 1230 | 0 | 60 | (Murphy et al. 2009) | | |
| **Europe** | Ireland | Pasture | 1838 | 27 | 67 | 2 | | 2465 | 666 | 1652 | 49 | 99 | (Murphy et al. 2009) | | |
| **Europe** | Ireland | Pasture | 1685 | 49 | 48 | 2 | | 2260 | 1107 | 1085 | 45 | 23 | (Murphy et al. 2009) | | |
| **Europe** | Ireland | Pasture | 1373 | 47 | 47 | 4 | | 1842 | 866 | 866 | 74 | 37 | (Murphy et al. 2009) | | |
| **Europe** | Ireland | Pasture | 1647 | 51 | 43 | 3 | | 2052 | 1047 | 883 | 62 | 62 | (Murphy et al. 2009) | | |
| **Europe** | Ireland | Pasture | 1832 | 30 | 63 | 2 | | 2250 | 675 | 1417 | 45 | 112 | (Murphy et al. 2009) | | |
| **Europe** | Ireland | Pasture | 1816 | 35 | 63 | 2 | | 2011 | 704 | 1267 | 40 | 0 | (Murphy et al. 2009) | | |
| **Europe** | Ireland | Pasture | 978 | 37 | 50 | 9 | | 1036 | 383 | 518 | 93 | 41 | (Murphy et al. 2009) | | |
| **Europe** | Ireland | Pasture | 1698 | 36 | 53 | 5 | | 1629 | 586 | 863 | 81 | 98 | (Murphy et al. 2009) | | |
| **Europe** | Ireland | Pasture | 1585 | 56 | 39 | 4 | | 1474 | 825 | 575 | 59 | 15 | (Murphy et al. 2009) | | |
| **Europe** | Ireland | Pasture | 1550 | 52 | 40 | 5 | | 268 | 139 | 107 | 13 | 8 | (Murphy et al. 2009) | | |
| **Europe** | Finland | Pasture | 978 | 32 | 50 | 2 | | 1371 | 439 | 686 | 33 | 214 | (Soinne et al. 2011) | | |
| **North America** | USA | Pasture | 548 | 14 | 54 | 12 | | 683 | 95 | 367 | 79 | 141 | (Lehmann et al. 2005) | | |
| **North America** | USA | Pasture | 1360 | 58 | 30 | 10 | | 2453 | 1423 | 736 | 245 | 49 | (Giles et al. 2015) | | |
| **Oceania** | NZ | Pasture | 1269 | 67 | 31 | 1 | | 1458 | 977 | 452 | 15 | 15 | (McDowell and Stewart 2006) | | |
| **Oceania** | NZ | Pasture | 945 | 43 | 49 | 1 | | 1429 | 615 | 700 | 14 | 100 | (McDowell and Stewart 2006) | | |
| **Oceania** | NZ | Pasture | 2405 | 58 | 48 | 2 | | 2703 | 1568 | 1298 | 54 | 0 | (McDowell and Stewart 2006) | | |
| **Oceania** | NZ | Pasture | 938 | 35 | 60 | 2 | | 1452 | 508 | 871 | 29 | 44 | (McDowell and Stewart 2006) | | |
| **Oceania** | NZ | Pasture | 2108 | 56 | 39 | 1 | | 2765 | 1549 | 1079 | 28 | 111 | (McDowell and Stewart 2006) | | |
| **Oceania** | NZ | Pasture | 1862 | 61 | 28 | 2 | | 1957 | 1194 | 548 | 39 | 176 | (McDowell et al. 2005) | | |
| **Oceania** | NZ | Pasture | 2746 | 63 | 25 | 2 | | 3427 | 2159 | 857 | 69 | 343 | (McDowell et al. 2005) | | |
| **Oceania** | NZ | Pasture | 682 | 51 | 36 | 4 | | 745 | 380 | 268 | 30 | 67 | (McDowell et al. 2005) | | |
| **Oceania** | NZ | Pasture | 1413 | 57 | 25 | 2 | | 1363 | 777 | 341 | 27 | 218 | (McDowell et al. 2005) | | |
| **Oceania** | NZ | Pasture | 1585 | 51 | 36 | 1 | | 1636 | 834 | 589 | 16 | 196 | (McDowell et al. 2005) | | |
| **Oceania** | NZ | Pasture | 1056 | 30 | 53 | 4 | | 1110 | 333 | 588 | 44 | 144 | (McDowell et al. 2005) | | |
| **Oceania** | NZ | Pasture | 1311 | 41 | 34 | 2 | | 1277 | 524 | 434 | 26 | 294 | (McDowell et al. 2005) | | |
| **Oceania** | NZ | Pasture | 487 | 46 | 50 | 1 | | 649 | 299 | 325 | 6 | 19 | (McDowell et al. 2005) | | |
| **Oceania** | NZ | Pasture | 690 | 57 | 28 | 1 | | 1196 | 682 | 335 | 12 | 167 | (McDowell et al. 2005) | | |
| **Oceania** | NZ | Pasture | 862 | 52 | 37 | 2 | | 1442 | 750 | 533 | 29 | 130 | (McDowell et al. 2005) | | |
| **Oceania** | NZ | Pasture | 904 | 49 | 45 | 3 | | 1433 | 702 | 645 | 43 | 43 | (McDowell et al. 2005) | | |
| **Oceania** | NZ | Pasture | 401 | 35 | 44 | 2 | | 619 | 217 | 272 | 12 | 118 | (McDowell et al. 2005) | | |
| **Oceania** | NZ | Pasture | 663 | 36 | 61 | 1 | | 950 | 342 | 580 | 10 | 19 | (McDowell et al. 2005) | | |
| **Oceania** | NZ | Pasture | 813 | 44 | 47 | 0 | | 1283 | 565 | 603 | 0 | 116 | (McDowell et al. 2005) | | |
| **Oceania** | NZ | Pasture | 1056 | 64 | 27 | 9 | | 1627 | 1041 | 439 | 146 | 0 | (McDowell et al. 2005) | | |
| **Oceania** | NZ | Pasture | 1291 | 55 | 32 | 15 | | 1935 | 1064 | 619 | 290 | 0 | (McDowell et al. 2005) | | |
| **Oceania** | NZ | Pasture | 707 | 40 | 60 | 0 | | 1018 | 407 | 611 | 0 | 0 | (McDowell et al. 2005) | | |
| **Oceania** | NZ | Pasture | 524 | 43 | 46 | 7 | | 815 | 350 | 375 | 57 | 33 | (McDowell et al. 2005) | | |
| **Oceania** | NZ | Pasture | 1236 | 40 | 42 | 6 | | 1827 | 731 | 767 | 110 | 219 | (McDowell et al. 2005) | | |
| **Oceania** | NZ | Pasture | 439 | 39 | 40 | 14 | | 709 | 277 | 284 | 99 | 50 | (McDowell et al. 2005) | | |
| **Oceania** | NZ | Pasture | 1255 | 64 | 24 | 17 | | 2016 | 1290 | 484 | 343 | 0 | (McDowell et al. 2005) | | |
| **Oceania** | NZ | Pasture | 560 | 64 | 27 | 18 | | 899 | 575 | 243 | 162 | 0 | (McDowell et al. 2005) | | |
| **Oceania** | NZ | Pasture | 116 | 32 | 30 | 8 | | 212 | 68 | 63 | 17 | 63 | (McDowell et al. 2005) | | |
| **Oceania** | NZ | Pasture | 317 | 44 | 42 | 5 | | 567 | 250 | 238 | 28 | 51 | (McDowell et al. 2005) | | |
| **Oceania** | NZ | Pasture | 999 | 59 | 36 | 1 | | 1390 | 820 | 501 | 14 | 56 | (McDowell et al. 2005) | | |
| **Oceania** | NZ | Pasture | 928 | 57 | 41 | 1 | | 1292 | 736 | 530 | 13 | 13 | (McDowell et al. 2005) | | |
| **Oceania** | Australia | Pasture | 460 | 43 | 53 | 5 | | 798 | 341 | 420 | 37 | 0 | (McDowell et al. 2005) | | |
| **Oceania** | Australia | Pasture | 13236 | 91 | 7 | 1 | | 20650 | 18730 | 1404 | 289 | 227 | (Doolette et al. 2009) | | |
| **Oceania** | Australia | Pasture | 1411 | 80 | 18 | 1 | | 2134 | 1707 | 382 | 26 | 19 | (Doolette et al. 2009) | | |
| **Oceania** | Australia | Pasture | 281 | 57 | 38 | 5 | | 479 | 273 | 180 | 24 | 2 | (Doolette et al. 2009) | | |
| **Oceania** | Australia | Pasture | 1241 | 64 | 30 | 4 | | 1936 | 1233 | 587 | 85 | 31 | (Doolette et al. 2009) | | |
| **Oceania** | Australia | Pasture | 867 | 73 | 24 | 2 | | 1301 | 949 | 316 | 21 | 16 | (Doolette et al. 2009) | | |
| **Oceania** | Australia | Pasture | 15111 | 90 | 8 | 1 | | 23576 | 21218 | 1886 | 236 | 236 | (Doolette et al. 2011) | | |
| **Oceania** | Australia | Pasture | 1576 | 76 | 21 | 1 | | 2384 | 1812 | 501 | 24 | 48 | (Doolette et al. 2011) | | |
| **Oceania** | Australia | Pasture | 257 | 52 | 43 | 1 | | 438 | 228 | 189 | 4 | 18 | (Doolette et al. 2011) | | |
| **Oceania** | Australia | Pasture | 1113 | 52 | 43 | 2 | | 1736 | 903 | 747 | 35 | 52 | (Doolette et al. 2011) | | |
| **Oceania** | Australia | Pasture | 1214 | 67 | 30 | 1 | | 1822 | 1221 | 547 | 18 | 36 | (Doolette et al. 2011) | | |
| **Oceania** | Australia | Pasture | 465 | 39 | 56 | 3 | | 504 | 196 | 282 | 15 | 10 | (Doolette et al. 2011) | | |
| **Oceania** | Australia | Pasture | 665 | 52 | 42 | 5 | | 720 | 375 | 302 | 36 | 7 | (Doolette et al. 2011) | | |
| **Oceania** | Australia | Pasture | 627 | 37 | 52 | 4 | | 1061 | 392 | 552 | 42 | 74 | (Doolette et al. 2011) | | |
| **Oceania** | Australia | Pasture | 1386 | 78 | 20 | 1 | | 2345 | 1829 | 469 | 23 | 23 | (Doolette et al. 2011) | | |
| **Oceania** | Australia | Pasture | 1106 | 46 | 47 | 4 | | 1128 | 519 | 530 | 45 | 34 | (Doolette et al. 2011) | | |
| **Oceania** | Australia | Pasture | 2526 | 82 | 15 | 2 | | 2576 | 2112 | 386 | 52 | 26 | (Doolette et al. 2011) | | |
| **Oceania** | Australia | Pasture | 1910 | 49 | 47 | 2 | | 2524 | 1237 | 1186 | 50 | 50 | (Doolette et al. 2011) | | |
| **Oceania** | Australia | Pasture | 2293 | 65 | 31 | 2 | | 3030 | 1970 | 939 | 61 | 61 | (Doolette et al. 2011) | | |
| **Oceania** | Australia | Pasture | 1299 | 66 | 31 | 1 | | 1504 | 993 | 466 | 18 | 27 | (Dougherty et al. 2007) | | |
| **Oceania** | Australia | Pasture | 1108 | 72 | 23 | 3 | | 1508 | 1086 | 345 | 38 | 39 | (Dougherty et al. 2007) | | |
| **Oceania** | Australia | Pasture | 545 | 51 | 43 | 4 | | 726 | 370 | 311 | 29 | 16 | (Dougherty et al. 2007) | | |
| **Oceania** | Australia | Pasture | 2450 | 41 | 46 | 7 | | 1382 | 571 | 632 | 102 | 77 | (George et al. 2006) | | |
| **South America** | Brazil | Pasture | 301 | 45 | 38 | 9 | | 505 | 227 | 193 | 46 | 39 | (Chapuis-Lardy et al. 2001) | | |
| **South America** | Colombia | Pasture | 358 | 6 | 53 | 37 | | 616 | 35 | 327 | 228 | 26 | (Guggenberger et al. 1996b) | | |
| **South America** | Colombia | Pasture | 382 | 5 | 53 | 36 | | 657 | 35 | 350 | 238 | 34 | (Guggenberger et al. 1996b) | | |

**Table S2.** Bootstaped populations (B=1000; R statistics) of soil P stocks in Kg ha^-1^ from table S1.

| **--------------Total P-------------** | | | | **Inorganic orthophosphate** | | | | **-------P Monoesters-----** | | | | **-------P Diesters----** | | | | **-------------Other P--------** | | | |
| --- | --- | --- | --- | --- | --- | --- | --- | --- | --- | --- | --- | --- | --- | --- | --- | --- | --- | --- | --- |
| 1964 | 1848 | 1607 | 1529 | 923 | 950 | 903 | 1111 | 576 | 649 | 570 | 583 | 61 | 65 | 67 | 60 | 88 | 108 | 121 | 93 |
| 1834 | 1799 | 1591 | 2037 | 937 | 867 | 993 | 933 | 610 | 607 | 592 | 561 | 74 | 73 | 81 | 60 | 81 | 105 | 105 | 109 |
| 1710 | 1849 | 1893 | 1867 | 1033 | 809 | 1092 | 868 | 594 | 578 | 582 | 616 | 62 | 63 | 79 | 62 | 85 | 90 | 98 | 118 |
| 2020 | 1853 | 1862 | 1810 | 932 | 979 | 940 | 1151 | 632 | 561 | 619 | 548 | 69 | 64 | 66 | 57 | 101 | 120 | 86 | 114 |
| 1825 | 1773 | 1614 | 1804 | 1199 | 1048 | 1092 | 929 | 547 | 586 | 641 | 598 | 57 | 58 | 70 | 55 | 97 | 99 | 107 | 86 |
| 1617 | 1656 | 1703 | 1965 | 1071 | 794 | 1178 | 1107 | 586 | 601 | 593 | 559 | 61 | 72 | 49 | 58 | 84 | 78 | 107 | 81 |
| 1885 | 1636 | 1725 | 1649 | 959 | 1037 | 833 | 1054 | 658 | 572 | 681 | 623 | 54 | 58 | 70 | 60 | 98 | 99 | 91 | 112 |
| 1781 | 1932 | 1632 | 1740 | 1092 | 1030 | 1058 | 827 | 558 | 619 | 547 | 556 | 64 | 59 | 66 | 51 | 79 | 83 | 129 | 89 |
| 1692 | 1650 | 2063 | 1856 | 966 | 957 | 1031 | 1034 | 599 | 568 | 645 | 548 | 55 | 51 | 64 | 69 | 103 | 79 | 101 | 80 |
| 1856 | 1530 | 1973 | 1736 | 867 | 1295 | 909 | 1010 | 590 | 606 | 618 | 557 | 65 | 62 | 65 | 68 | 90 | 108 | 95 | 96 |
| 1739 | 1626 | 1761 | 1768 | 984 | 1094 | 943 | 1022 | 593 | 562 | 567 | 547 | 69 | 71 | 62 | 64 | 112 | 84 | 70 | 86 |
| 1574 | 1781 | 1815 | 1591 | 1029 | 1083 | 1112 | 933 | 588 | 638 | 589 | 586 | 67 | 75 | 79 | 74 | 100 | 101 | 102 | 74 |
| 1652 | 1973 | 1894 | 2137 | 987 | 891 | 955 | 1010 | 530 | 556 | 619 | 582 | 62 | 58 | 67 | 64 | 101 | 84 | 94 | 88 |
| 1657 | 1568 | 1903 | 1864 | 1182 | 924 | 1024 | 970 | 560 | 583 | 558 | 593 | 60 | 75 | 57 | 56 | 93 | 95 | 114 | 100 |
| 1657 | 1911 | 1677 | 1852 | 816 | 1022 | 1133 | 813 | 555 | 570 | 607 | 586 | 69 | 52 | 56 | 58 | 126 | 96 | 103 | 113 |
| 1764 | 1653 | 1527 | 1787 | 920 | 861 | 917 | 1069 | 588 | 545 | 662 | 606 | 67 | 67 | 58 | 67 | 103 | 100 | 71 | 106 |
| 1756 | 1779 | 1616 | 1623 | 1125 | 847 | 843 | 905 | 589 | 618 | 593 | 577 | 70 | 71 | 60 | 61 | 80 | 80 | 116 | 88 |
| 1662 | 2056 | 1663 | 1685 | 853 | 1016 | 1021 | 848 | 596 | 583 | 601 | 571 | 67 | 63 | 63 | 70 | 78 | 117 | 92 | 80 |
| 1959 | 1941 | 1560 | 1683 | 965 | 998 | 1036 | 848 | 559 | 572 | 570 | 615 | 68 | 63 | 51 | 65 | 88 | 99 | 109 | 87 |
| 1899 | 1928 | 1803 | 1853 | 1260 | 881 | 890 | 868 | 594 | 575 | 593 | 611 | 62 | 75 | 64 | 68 | 98 | 70 | 86 | 85 |
| 1636 | 1841 | 1838 | 1909 | 1287 | 991 | 1032 | 791 | 606 | 598 | 598 | 597 | 63 | 57 | 73 | 69 | 99 | 94 | 96 | 121 |
| 1682 | 1711 | 1464 | 1652 | 937 | 938 | 865 | 1218 | 563 | 594 | 599 | 526 | 74 | 69 | 63 | 71 | 92 | 86 | 113 | 68 |
| 1921 | 1860 | 1741 | 1912 | 1125 | 909 | 1113 | 1020 | 551 | 524 | 604 | 657 | 67 | 62 | 52 | 54 | 84 | 85 | 87 | 96 |
| 1669 | 1797 | 1877 | 1826 | 1131 | 962 | 988 | 967 | 614 | 620 | 573 | 637 | 68 | 53 | 64 | 54 | 104 | 89 | 119 | 111 |
| 1984 | 1704 | 1581 | 1897 | 958 | 1041 | 1181 | 867 | 563 | 560 | 566 | 522 | 57 | 61 | 76 | 81 | 96 | 100 | 116 | 91 |
| 1535 | 1602 | 1838 | 1816 | 1048 | 949 | 819 | 1117 | 576 | 626 | 581 | 588 | 64 | 60 | 65 | 59 | 84 | 83 | 118 | 108 |
| 1733 | 1872 | 1598 | 1807 | 992 | 877 | 1050 | 1085 | 639 | 621 | 611 | 546 | 54 | 56 | 55 | 55 | 99 | 93 | 100 | 78 |
| 1867 | 1842 | 1610 | 1428 | 1147 | 834 | 993 | 935 | 565 | 591 | 604 | 532 | 70 | 62 | 56 | 77 | 90 | 84 | 78 | 90 |
| 1837 | 1581 | 1779 | 1584 | 1131 | 851 | 963 | 930 | 575 | 598 | 612 | 541 | 63 | 63 | 73 | 76 | 123 | 102 | 96 | 99 |
| 1551 | 1701 | 2061 | 1930 | 1228 | 1043 | 987 | 973 | 604 | 529 | 559 | 636 | 66 | 52 | 65 | 75 | 96 | 100 | 114 | 78 |
| 1770 | 1659 | 1830 | 1601 | 854 | 982 | 1050 | 1049 | 602 | 633 | 595 | 568 | 59 | 66 | 67 | 65 | 95 | 74 | 84 | 83 |
| 1719 | 1867 | 1959 | 1618 | 858 | 1036 | 1127 | 1146 | 594 | 632 | 580 | 629 | 62 | 67 | 63 | 58 | 107 | 100 | 65 | 108 |
| 2030 | 1898 | 1655 | 1957 | 1004 | 904 | 1078 | 1110 | 580 | 593 | 559 | 528 | 67 | 64 | 66 | 63 | 92 | 78 | 83 | 95 |
| 1612 | 1593 | 1620 | 1775 | 1015 | 987 | 904 | 1214 | 563 | 542 | 626 | 578 | 79 | 58 | 58 | 63 | 92 | 96 | 115 | 92 |
| 2020 | 1914 | 2136 | 1622 | 764 | 922 | 1021 | 1002 | 587 | 577 | 628 | 530 | 71 | 62 | 53 | 58 | 105 | 86 | 95 | 80 |
| 2169 | 1714 | 1819 | 1526 | 1049 | 1039 | 991 | 987 | 625 | 566 | 601 | 621 | 67 | 70 | 53 | 73 | 107 | 84 | 85 | 96 |
| 1725 | 1767 | 1793 | 1920 | 976 | 831 | 1057 | 1124 | 590 | 530 | 565 | 610 | 53 | 68 | 61 | 60 | 80 | 82 | 103 | 86 |
| 1705 | 1618 | 1683 | 1646 | 1134 | 1004 | 913 | 941 | 608 | 557 | 595 | 609 | 49 | 62 | 59 | 72 | 100 | 79 | 72 | 73 |
| 1906 | 1540 | 1822 | 1983 | 976 | 944 | 1041 | 813 | 568 | 591 | 668 | 591 | 66 | 66 | 65 | 60 | 87 | 116 | 94 | 106 |
| 1628 | 2124 | 1879 | 1672 | 963 | 900 | 981 | 1013 | 561 | 610 | 635 | 586 | 69 | 69 | 67 | 62 | 93 | 88 | 91 | 138 |
| 1688 | 1656 | 1963 | 1658 | 836 | 1033 | 1014 | 840 | 571 | 600 | 601 | 622 | 62 | 67 | 65 | 55 | 101 | 72 | 106 | 93 |
| 1977 | 1904 | 1951 | 1506 | 1173 | 857 | 941 | 879 | 601 | 635 | 634 | 597 | 67 | 69 | 63 | 65 | 110 | 106 | 121 | 105 |
| 2139 | 1661 | 2036 | 1877 | 921 | 1035 | 1039 | 964 | 579 | 608 | 562 | 621 | 64 | 64 | 66 | 74 | 106 | 86 | 136 | 90 |
| 1849 | 1721 | 1622 | 1836 | 1143 | 1014 | 1090 | 1145 | 571 | 578 | 512 | 549 | 61 | 52 | 55 | 85 | 99 | 100 | 73 | 93 |
| 1900 | 1902 | 1724 | 2131 | 940 | 1015 | 1019 | 1125 | 619 | 547 | 597 | 575 | 64 | 62 | 57 | 66 | 126 | 77 | 95 | 123 |
| 1764 | 1760 | 1725 | 1676 | 1160 | 1061 | 1085 | 1090 | 602 | 563 | 607 | 572 | 68 | 67 | 76 | 65 | 105 | 92 | 95 | 83 |
| 1920 | 1651 | 1758 | 1823 | 1216 | 983 | 1026 | 928 | 644 | 620 | 595 | 514 | 66 | 62 | 72 | 56 | 84 | 77 | 73 | 132 |
| 1814 | 1786 | 1644 | 1784 | 949 | 1248 | 1086 | 899 | 591 | 552 | 562 | 547 | 55 | 69 | 49 | 71 | 135 | 99 | 78 | 130 |
| 1672 | 1603 | 1486 | 1638 | 809 | 971 | 880 | 1200 | 578 | 567 | 585 | 576 | 71 | 71 | 65 | 54 | 108 | 83 | 108 | 99 |
| 1693 | 1663 | 1706 | 1814 | 942 | 1006 | 971 | 997 | 641 | 519 | 615 | 566 | 71 | 64 | 69 | 57 | 88 | 81 | 94 | 73 |
| 1976 | 1733 | 1631 | 1697 | 957 | 1012 | 1221 | 1006 | 600 | 665 | 615 | 597 | 62 | 62 | 62 | 65 | 101 | 73 | 99 | 75 |
| 1619 | 1662 | 2112 | 1705 | 975 | 951 | 957 | 1000 | 599 | 569 | 588 | 585 | 65 | 60 | 50 | 64 | 101 | 90 | 87 | 80 |
| 1683 | 1874 | 1843 | 1648 | 1140 | 913 | 924 | 1071 | 600 | 592 | 553 | 575 | 51 | 61 | 63 | 60 | 106 | 96 | 84 | 92 |
| 1738 | 1914 | 1839 | 1575 | 1122 | 990 | 925 | 960 | 581 | 600 | 616 | 559 | 76 | 59 | 53 | 71 | 96 | 104 | 96 | 104 |
| 1775 | 1731 | 1795 | 1566 | 1253 | 984 | 1139 | 948 | 570 | 551 | 635 | 534 | 58 | 60 | 57 | 63 | 103 | 106 | 96 | 74 |
| 1752 | 1582 | 1942 | 1884 | 1166 | 906 | 892 | 1293 | 566 | 551 | 546 | 555 | 68 | 71 | 82 | 59 | 104 | 105 | 115 | 84 |
| 1846 | 1770 | 1784 | 1728 | 956 | 1002 | 1109 | 1104 | 586 | 624 | 626 | 603 | 65 | 61 | 55 | 78 | 75 | 91 | 109 | 119 |
| 1603 | 1596 | 1652 | 2003 | 943 | 1014 | 866 | 1040 | 625 | 577 | 600 | 569 | 69 | 65 | 58 | 68 | 120 | 84 | 95 | 96 |
| 1727 | 1680 | 1841 | 1672 | 842 | 1145 | 958 | 1089 | 641 | 596 | 531 | 565 | 53 | 64 | 56 | 53 | 103 | 114 | 95 | 73 |
| 1892 | 1592 | 1651 | 1912 | 851 | 994 | 1423 | 851 | 666 | 604 | 577 | 611 | 73 | 69 | 62 | 53 | 75 | 81 | 93 | 116 |
| 1784 | 1607 | 1727 | 1936 | 1200 | 1143 | 976 | 965 | 647 | 653 | 554 | 610 | 65 | 77 | 70 | 77 | 127 | 114 | 78 | 96 |
| 1553 | 1655 | 1681 | 1768 | 1273 | 956 | 1373 | 803 | 628 | 562 | 592 | 552 | 51 | 57 | 63 | 64 | 113 | 102 | 75 | 104 |
| 1889 | 1768 | 1561 | 1674 | 1114 | 986 | 989 | 1131 | 581 | 583 | 601 | 586 | 69 | 61 | 61 | 53 | 97 | 95 | 112 | 106 |
| 1748 | 1712 | 1865 | 1772 | 1270 | 842 | 1181 | 848 | 597 | 622 | 596 | 615 | 73 | 75 | 68 | 56 | 81 | 103 | 75 | 86 |
| 1966 | 1924 | 1615 | 1717 | 995 | 897 | 1173 | 1046 | 563 | 552 | 569 | 565 | 65 | 61 | 58 | 65 | 90 | 84 | 88 | 69 |
| 1840 | 1914 | 1539 | 1748 | 1061 | 831 | 915 | 1041 | 550 | 578 | 538 | 587 | 76 | 72 | 62 | 59 | 89 | 78 | 81 | 85 |
| 1710 | 1900 | 1856 | 1822 | 1020 | 1120 | 855 | 1004 | 524 | 539 | 562 | 628 | 58 | 71 | 58 | 67 | 93 | 109 | 102 | 91 |
| 1795 | 1642 | 1896 | 1495 | 874 | 1041 | 918 | 1037 | 615 | 612 | 620 | 592 | 58 | 59 | 56 | 53 | 78 | 102 | 89 | 97 |
| 1798 | 1607 | 1761 | 1750 | 962 | 910 | 790 | 939 | 593 | 573 | 583 | 577 | 63 | 62 | 62 | 47 | 95 | 96 | 78 | 82 |
| 1995 | 1675 | 1740 | 1626 | 1027 | 1016 | 1019 | 1206 | 563 | 582 | 632 | 540 | 55 | 63 | 71 | 58 | 110 | 105 | 112 | 108 |
| 1714 | 1666 | 1667 | 1935 | 863 | 953 | 1016 | 1126 | 575 | 558 | 546 | 617 | 54 | 69 | 72 | 68 | 122 | 101 | 87 | 78 |
| 1740 | 1688 | 1706 | 1882 | 1025 | 1193 | 849 | 1120 | 549 | 534 | 589 | 584 | 64 | 61 | 55 | 53 | 79 | 88 | 104 | 89 |
| 1644 | 1899 | 1763 | 1770 | 924 | 1109 | 1210 | 860 | 695 | 591 | 649 | 526 | 62 | 71 | 67 | 71 | 93 | 92 | 59 | 118 |
| 1907 | 1833 | 1649 | 1787 | 1135 | 1048 | 980 | 1312 | 547 | 585 | 583 | 611 | 60 | 62 | 57 | 66 | 92 | 83 | 76 | 102 |
| 1853 | 1715 | 1603 | 1772 | 811 | 936 | 1023 | 904 | 588 | 595 | 584 | 550 | 72 | 53 | 72 | 74 | 82 | 93 | 107 | 113 |
| 1721 | 1815 | 1735 | 1519 | 841 | 1109 | 1011 | 1032 | 601 | 585 | 551 | 543 | 59 | 67 | 57 | 64 | 102 | 119 | 91 | 95 |
| 1814 | 1721 | 1876 | 1697 | 920 | 1212 | 1122 | 886 | 592 | 630 | 561 | 557 | 68 | 65 | 60 | 60 | 78 | 90 | 134 | 88 |
| 1642 | 1648 | 1766 | 1870 | 1296 | 880 | 908 | 1123 | 595 | 577 | 614 | 572 | 65 | 67 | 63 | 75 | 90 | 81 | 90 | 98 |
| 1916 | 1783 | 1685 | 1705 | 1015 | 991 | 979 | 939 | 581 | 546 | 589 | 553 | 72 | 71 | 59 | 63 | 78 | 90 | 103 | 107 |
| 1802 | 1773 | 1620 | 1873 | 1178 | 915 | 853 | 1193 | 552 | 568 | 590 | 574 | 78 | 60 | 68 | 59 | 91 | 98 | 97 | 73 |
| 1634 | 1704 | 1874 | 1936 | 1185 | 907 | 1165 | 1071 | 612 | 600 | 575 | 558 | 55 | 60 | 54 | 76 | 105 | 104 | 94 | 106 |
| 1984 | 1714 | 1900 | 1620 | 877 | 984 | 842 | 1072 | 607 | 557 | 552 | 610 | 52 | 63 | 66 | 70 | 95 | 97 | 95 | 130 |
| 1750 | 1770 | 1806 | 1646 | 966 | 843 | 1059 | 1248 | 563 | 574 | 606 | 632 | 59 | 64 | 68 | 62 | 94 | 76 | 110 | 90 |
| 1711 | 1645 | 1697 | 1873 | 984 | 1206 | 1011 | 1069 | 575 | 576 | 652 | 559 | 71 | 76 | 70 | 77 | 81 | 122 | 109 | 98 |
| 1770 | 1806 | 1866 | 1760 | 805 | 1202 | 1158 | 877 | 634 | 546 | 547 | 576 | 68 | 68 | 69 | 72 | 84 | 99 | 115 | 92 |
| 1753 | 1577 | 1671 | 1916 | 1083 | 964 | 1019 | 1136 | 578 | 573 | 575 | 562 | 71 | 74 | 68 | 60 | 113 | 91 | 95 | 101 |
| 1822 | 1527 | 1795 | 1749 | 1093 | 1142 | 1130 | 844 | 575 | 580 | 625 | 548 | 54 | 64 | 64 | 68 | 91 | 129 | 82 | 101 |
| 1686 | 1582 | 1679 | 1644 | 922 | 1046 | 1029 | 1107 | 583 | 547 | 603 | 576 | 60 | 61 | 59 | 53 | 93 | 97 | 96 | 82 |
| 1823 | 1741 | 1648 | 1709 | 833 | 1071 | 914 | 887 | 595 | 562 | 599 | 589 | 60 | 67 | 65 | 59 | 85 | 98 | 102 | 91 |
| 1769 | 1814 | 1709 | 1767 | 1079 | 971 | 1031 | 1008 | 533 | 569 | 587 | 631 | 67 | 58 | 67 | 49 | 90 | 95 | 104 | 111 |
| 1915 | 1766 | 1692 | 1946 | 878 | 931 | 1009 | 944 | 610 | 617 | 540 | 572 | 68 | 69 | 75 | 61 | 113 | 87 | 88 | 109 |
| 1780 | 1723 | 1728 | 1533 | 982 | 1167 | 1020 | 1032 | 598 | 669 | 550 | 647 | 64 | 72 | 69 | 61 | 85 | 105 | 110 | 82 |
| 1672 | 1875 | 1754 | 1689 | 999 | 1062 | 1078 | 1001 | 580 | 588 | 640 | 590 | 82 | 57 | 62 | 68 | 118 | 89 | 85 | 108 |
| 1622 | 1664 | 1599 | 1810 | 971 | 1058 | 910 | 986 | 598 | 602 | 600 | 609 | 65 | 77 | 63 | 65 | 97 | 107 | 111 | 96 |
| 1807 | 1918 | 1935 | 1690 | 1139 | 811 | 849 | 1330 | 580 | 564 | 606 | 571 | 73 | 73 | 58 | 72 | 80 | 120 | 94 | 75 |
| 1915 | 1726 | 1629 | 1790 | 925 | 893 | 944 | 985 | 591 | 620 | 601 | 578 | 73 | 74 | 77 | 59 | 105 | 77 | 86 | 88 |
| 1550 | 1790 | 1556 | 1821 | 1042 | 1010 | 1084 | 949 | 566 | 592 | 588 | 572 | 64 | 67 | 75 | 60 | 106 | 72 | 96 | 88 |
| 1505 | 1676 | 2024 | 1621 | 852 | 979 | 915 | 1007 | 562 | 601 | 563 | 557 | 56 | 79 | 66 | 60 | 85 | 114 | 118 | 105 |
| 1768 | 1782 | 1878 | 1643 | 845 | 1474 | 981 | 800 | 591 | 594 | 636 | 545 | 70 | 71 | 58 | 60 | 93 | 108 | 86 | 100 |
| 1622 | 1828 | 1748 | 1981 | 1049 | 945 | 775 | 1161 | 621 | 583 | 589 | 675 | 60 | 73 | 76 | 65 | 108 | 76 | 92 | 96 |
| 1623 | 1751 | 1808 | 1502 | 982 | 974 | 1204 | 1211 | 562 | 567 | 579 | 594 | 60 | 67 | 56 | 64 | 91 | 73 | 100 | 106 |
| 1844 | 1708 | 1907 | 1803 | 1094 | 1132 | 993 | 889 | 612 | 569 | 632 | 591 | 76 | 70 | 67 | 56 | 107 | 91 | 85 | 137 |
| 1739 | 1711 | 1811 | 1728 | 954 | 1259 | 937 | 1117 | 586 | 609 | 576 | 594 | 60 | 69 | 59 | 56 | 96 | 94 | 111 | 86 |
| 1698 | 1498 | 1719 | 2024 | 1146 | 1016 | 969 | 954 | 589 | 567 | 627 | 652 | 65 | 58 | 66 | 74 | 76 | 84 | 87 | 93 |
| 1693 | 1829 | 1614 | 1818 | 911 | 998 | 982 | 995 | 608 | 593 | 559 | 563 | 49 | 75 | 68 | 73 | 92 | 81 | 79 | 93 |
| 1887 | 1552 | 1819 | 1703 | 1040 | 1075 | 1391 | 896 | 605 | 604 | 550 | 550 | 78 | 68 | 49 | 72 | 118 | 79 | 107 | 81 |
| 1814 | 1874 | 1734 | 1867 | 1062 | 961 | 1089 | 926 | 625 | 578 | 605 | 589 | 70 | 62 | 56 | 69 | 104 | 77 | 96 | 86 |
| 1784 | 1735 | 1613 | 2086 | 1009 | 1113 | 869 | 850 | 610 | 541 | 544 | 582 | 56 | 50 | 61 | 63 | 106 | 111 | 86 | 112 |
| 1653 | 2075 | 1748 | 1692 | 1095 | 894 | 1066 | 1014 | 629 | 555 | 557 | 540 | 67 | 60 | 65 | 64 | 91 | 98 | 89 | 87 |
| 1828 | 1830 | 1789 | 1840 | 1041 | 861 | 1056 | 1093 | 580 | 559 | 564 | 578 | 54 | 65 | 74 | 64 | 92 | 98 | 76 | 80 |
| 1998 | 1793 | 1805 | 1729 | 1058 | 881 | 1022 | 1026 | 538 | 550 | 642 | 549 | 69 | 52 | 59 | 65 | 108 | 72 | 117 | 97 |
| 1716 | 1856 | 1767 | 1619 | 910 | 907 | 965 | 1031 | 604 | 623 | 556 | 569 | 65 | 50 | 61 | 64 | 87 | 104 | 92 | 92 |
| 1611 | 1736 | 1784 | 1759 | 1078 | 1127 | 789 | 1141 | 552 | 601 | 591 | 611 | 64 | 73 | 61 | 51 | 97 | 85 | 97 | 86 |
| 1786 | 1659 | 1831 | 2274 | 1399 | 957 | 1224 | 864 | 577 | 563 | 584 | 550 | 60 | 64 | 60 | 75 | 96 | 88 | 101 | 71 |
| 1662 | 1649 | 1636 | 1719 | 883 | 938 | 1023 | 938 | 644 | 626 | 545 | 598 | 78 | 61 | 62 | 74 | 106 | 108 | 80 | 79 |
| 1864 | 1621 | 1745 | 1732 | 1049 | 1011 | 950 | 895 | 629 | 593 | 539 | 558 | 63 | 62 | 64 | 68 | 75 | 92 | 111 | 90 |
| 1662 | 1715 | 1590 | 1799 | 1036 | 1008 | 954 | 939 | 593 | 575 | 605 | 598 | 64 | 64 | 64 | 62 | 101 | 85 | 94 | 82 |
| 1673 | 1810 | 1482 | 1625 | 1138 | 1059 | 1236 | 1171 | 576 | 563 | 580 | 595 | 68 | 79 | 58 | 75 | 90 | 105 | 82 | 92 |
| 1687 | 1610 | 1676 | 1954 | 980 | 1037 | 1272 | 1016 | 573 | 579 | 567 | 552 | 52 | 59 | 62 | 51 | 95 | 97 | 95 | 113 |
| 1733 | 1657 | 1872 | 1860 | 855 | 923 | 1033 | 1034 | 579 | 586 | 591 | 564 | 65 | 69 | 60 | 62 | 76 | 105 | 103 | 93 |
| 1834 | 1995 | 1721 | 1752 | 936 | 1081 | 1037 | 1026 | 612 | 552 | 673 | 618 | 60 | 66 | 55 | 57 | 83 | 106 | 75 | 89 |
| 1605 | 1888 | 1608 | 1867 | 1132 | 1093 | 975 | 978 | 564 | 561 | 607 | 602 | 58 | 70 | 61 | 68 | 90 | 110 | 103 | 86 |
| 1521 | 1688 | 1702 | 1788 | 964 | 1090 | 1198 | 959 | 566 | 580 | 611 | 588 | 68 | 66 | 53 | 67 | 121 | 100 | 91 | 94 |
| 1602 | 1776 | 1618 | 1745 | 773 | 1152 | 982 | 914 | 559 | 548 | 576 | 568 | 68 | 63 | 62 | 63 | 92 | 101 | 102 | 95 |
| 1775 | 1828 | 1830 | 1806 | 1006 | 964 | 823 | 839 | 669 | 547 | 593 | 626 | 54 | 66 | 75 | 65 | 106 | 84 | 101 | 76 |
| 1919 | 1663 | 1622 | 1823 | 963 | 959 | 1118 | 1071 | 596 | 594 | 520 | 522 | 55 | 67 | 65 | 57 | 65 | 105 | 86 | 85 |
| 1763 | 1817 | 1714 | 1760 | 960 | 1070 | 1092 | 904 | 538 | 529 | 579 | 619 | 68 | 66 | 63 | 61 | 118 | 121 | 99 | 92 |
| 1785 | 1755 | 1600 | 1882 | 1011 | 959 | 835 | 1098 | 518 | 589 | 568 | 579 | 60 | 67 | 72 | 61 | 100 | 80 | 102 | 102 |
| 1500 | 1636 | 1838 | 1853 | 1186 | 955 | 1069 | 1102 | 568 | 597 | 614 | 579 | 57 | 74 | 61 | 64 | 112 | 76 | 116 | 99 |
| 1798 | 1787 | 1910 | 1665 | 985 | 1001 | 986 | 1059 | 615 | 596 | 598 | 545 | 57 | 66 | 71 | 84 | 106 | 98 | 95 | 107 |
| 1909 | 1853 | 1868 | 1802 | 1207 | 968 | 933 | 931 | 545 | 556 | 570 | 589 | 70 | 62 | 64 | 72 | 81 | 83 | 94 | 81 |
| 1931 | 1513 | 1896 | 1639 | 914 | 1026 | 953 | 976 | 563 | 640 | 569 | 622 | 61 | 62 | 51 | 65 | 101 | 83 | 97 | 92 |
| 1747 | 2160 | 1856 | 1842 | 994 | 878 | 1034 | 1135 | 562 | 626 | 552 | 622 | 70 | 78 | 54 | 69 | 95 | 70 | 131 | 106 |
| 1890 | 2050 | 1616 | 1713 | 1050 | 939 | 1021 | 953 | 572 | 608 | 576 | 618 | 58 | 60 | 59 | 51 | 122 | 101 | 96 | 82 |
| 1582 | 1884 | 2024 | 1952 | 1116 | 1040 | 1135 | 1076 | 605 | 598 | 589 | 610 | 79 | 55 | 57 | 52 | 96 | 105 | 76 | 88 |
| 1843 | 1884 | 1584 | 1834 | 892 | 907 | 885 | 937 | 558 | 626 | 571 | 588 | 64 | 64 | 69 | 58 | 95 | 76 | 115 | 94 |
| 1721 | 1919 | 1770 | 1904 | 986 | 830 | 1164 | 1032 | 606 | 578 | 559 | 651 | 57 | 67 | 57 | 74 | 102 | 91 | 97 | 92 |
| 1721 | 1880 | 1696 | 1529 | 955 | 889 | 1044 | 1136 | 607 | 560 | 666 | 543 | 64 | 66 | 56 | 63 | 98 | 85 | 116 | 85 |
| 1835 | 1721 | 1818 | 1946 | 1019 | 1119 | 1100 | 881 | 643 | 583 | 565 | 538 | 75 | 67 | 61 | 67 | 112 | 92 | 80 | 126 |
| 1702 | 1716 | 2084 | 1702 | 1111 | 1130 | 1071 | 1026 | 681 | 558 | 604 | 586 | 62 | 70 | 65 | 72 | 106 | 80 | 87 | 121 |
| 1657 | 1670 | 1819 | 1756 | 954 | 1171 | 1264 | 1147 | 535 | 614 | 575 | 573 | 69 | 67 | 61 | 63 | 101 | 90 | 101 | 85 |
| 1779 | 1473 | 1796 | 1769 | 942 | 1013 | 894 | 987 | 593 | 610 | 603 | 593 | 62 | 57 | 57 | 59 | 79 | 84 | 99 | 79 |
| 1789 | 1700 | 1717 | 1673 | 1002 | 1002 | 1112 | 977 | 564 | 595 | 573 | 607 | 59 | 76 | 53 | 65 | 115 | 115 | 86 | 97 |
| 2253 | 1854 | 1684 | 1697 | 883 | 1126 | 1006 | 978 | 620 | 590 | 614 | 569 | 56 | 73 | 71 | 67 | 109 | 82 | 84 | 97 |
| 1790 | 1622 | 1686 | 1680 | 782 | 1155 | 1239 | 1183 | 548 | 584 | 565 | 547 | 64 | 52 | 55 | 60 | 101 | 85 | 100 | 93 |
| 1782 | 1833 | 1868 | 2006 | 1144 | 1016 | 904 | 989 | 651 | 592 | 554 | 566 | 62 | 64 | 59 | 66 | 122 | 106 | 104 | 103 |
| 1727 | 1823 | 1616 | 1905 | 949 | 974 | 1077 | 875 | 593 | 620 | 664 | 589 | 65 | 82 | 66 | 61 | 105 | 89 | 92 | 98 |
| 1857 | 1630 | 1702 | 1685 | 949 | 1035 | 956 | 983 | 517 | 618 | 569 | 588 | 58 | 48 | 60 | 58 | 90 | 92 | 104 | 66 |
| 1600 | 1776 | 1750 | 1871 | 944 | 974 | 957 | 966 | 574 | 618 | 573 | 593 | 66 | 83 | 66 | 54 | 81 | 95 | 110 | 99 |
| 1669 | 1708 | 1987 | 1699 | 1180 | 990 | 1038 | 874 | 588 | 555 | 615 | 620 | 64 | 69 | 66 | 62 | 94 | 109 | 94 | 104 |
| 1695 | 1875 | 1933 | 1585 | 1029 | 1038 | 959 | 991 | 637 | 584 | 601 | 560 | 74 | 68 | 59 | 64 | 92 | 110 | 108 | 87 |
| 1720 | 1673 | 1930 | 1760 | 886 | 1053 | 807 | 1194 | 587 | 545 | 601 | 590 | 63 | 72 | 63 | 74 | 96 | 102 | 83 | 101 |
| 1659 | 1697 | 1779 | 1690 | 994 | 1118 | 869 | 1018 | 529 | 597 | 577 | 570 | 56 | 66 | 61 | 66 | 99 | 91 | 103 | 112 |
| 1915 | 1692 | 1539 | 1774 | 1074 | 870 | 940 | 983 | 560 | 665 | 614 | 595 | 65 | 66 | 68 | 47 | 96 | 115 | 107 | 94 |
| 1795 | 1936 | 2004 | 1765 | 1056 | 1162 | 1010 | 892 | 590 | 625 | 589 | 574 | 61 | 60 | 64 | 60 | 115 | 88 | 92 | 94 |
| 1505 | 1724 | 2015 | 1863 | 1067 | 940 | 1252 | 1296 | 594 | 589 | 611 | 591 | 65 | 76 | 92 | 58 | 93 | 105 | 113 | 97 |
| 1748 | 1697 | 1834 | 1803 | 998 | 1038 | 952 | 975 | 564 | 583 | 574 | 650 | 64 | 55 | 57 | 57 | 89 | 106 | 83 | 94 |
| 1656 | 1893 | 1906 | 1709 | 904 | 1165 | 1142 | 982 | 591 | 548 | 606 | 584 | 79 | 59 | 66 | 62 | 97 | 101 | 105 | 109 |
| 1943 | 1654 | 2003 | 1682 | 868 | 922 | 910 | 1193 | 562 | 618 | 613 | 557 | 70 | 57 | 77 | 66 | 110 | 99 | 87 | 90 |
| 1580 | 1797 | 1774 | 1681 | 951 | 953 | 1132 | 988 | 548 | 601 | 643 | 578 | 71 | 60 | 76 | 82 | 98 | 101 | 103 | 111 |
| 1627 | 1638 | 1651 | 1708 | 887 | 1299 | 1034 | 828 | 547 | 546 | 595 | 598 | 72 | 64 | 55 | 59 | 106 | 70 | 108 | 104 |
| 1773 | 2011 | 1765 | 1736 | 966 | 973 | 1117 | 1077 | 610 | 549 | 616 | 588 | 59 | 69 | 68 | 56 | 105 | 79 | 110 | 83 |
| 1857 | 1870 | 1688 | 2010 | 1005 | 858 | 859 | 1155 | 599 | 554 | 601 | 530 | 72 | 66 | 56 | 61 | 73 | 94 | 103 | 84 |
| 1743 | 1638 | 1653 | 1681 | 1101 | 862 | 883 | 1007 | 560 | 611 | 609 | 575 | 77 | 63 | 70 | 60 | 88 | 100 | 101 | 109 |
| 1683 | 1949 | 1589 | 1966 | 918 | 1193 | 1143 | 962 | 543 | 602 | 571 | 643 | 65 | 67 | 68 | 64 | 98 | 107 | 111 | 109 |
| 1636 | 1921 | 1842 | 1830 | 855 | 987 | 1021 | 887 | 543 | 550 | 609 | 584 | 52 | 64 | 70 | 60 | 93 | 117 | 80 | 90 |
| 1713 | 1842 | 1886 | 1894 | 921 | 969 | 1021 | 882 | 689 | 599 | 588 | 627 | 57 | 67 | 63 | 54 | 83 | 101 | 100 | 106 |
| 1593 | 1897 | 1548 | 1690 | 1076 | 1020 | 914 | 921 | 562 | 645 | 590 | 604 | 74 | 70 | 74 | 67 | 100 | 117 | 113 | 109 |
| 1625 | 1764 | 2078 | 1887 | 956 | 1103 | 1079 | 981 | 579 | 642 | 596 | 573 | 61 | 76 | 50 | 61 | 78 | 94 | 80 | 89 |
| 1709 | 1863 | 1732 | 1730 | 945 | 1059 | 1099 | 953 | 572 | 610 | 608 | 590 | 45 | 76 | 65 | 66 | 101 | 84 | 109 | 104 |
| 1761 | 1982 | 1734 | 1644 | 1048 | 1016 | 1113 | 917 | 568 | 632 | 547 | 632 | 80 | 61 | 66 | 71 | 93 | 99 | 101 | 90 |
| 1820 | 1841 | 1897 | 1809 | 1178 | 1062 | 1111 | 1209 | 655 | 606 | 590 | 570 | 59 | 55 | 67 | 61 | 89 | 87 | 99 | 98 |
| 1790 | 1698 | 1554 | 1777 | 911 | 1076 | 955 | 917 | 593 | 569 | 635 | 607 | 63 | 75 | 57 | 55 | 80 | 114 | 118 | 103 |
| 1714 | 1787 | 1578 | 2135 | 991 | 1064 | 957 | 1143 | 595 | 607 | 570 | 657 | 65 | 43 | 74 | 54 | 82 | 108 | 111 | 114 |
| 1802 | 1877 | 1567 | 1867 | 1181 | 948 | 809 | 1058 | 577 | 573 | 617 | 540 | 53 | 54 | 58 | 63 | 114 | 103 | 95 | 117 |
| 1639 | 1726 | 1684 | 1798 | 1010 | 1000 | 868 | 904 | 587 | 599 | 605 | 597 | 51 | 74 | 70 | 58 | 106 | 117 | 87 | 103 |
| 1672 | 2087 | 1707 | 1691 | 966 | 979 | 1033 | 1177 | 569 | 596 | 588 | 543 | 55 | 63 | 58 | 52 | 91 | 85 | 72 | 84 |
| 1751 | 1709 | 1710 | 1833 | 918 | 1004 | 1022 | 1075 | 571 | 633 | 649 | 672 | 66 | 58 | 52 | 61 | 84 | 84 | 105 | 82 |
| 1639 | 1655 | 1861 | 1822 | 1043 | 1178 | 885 | 947 | 589 | 611 | 617 | 566 | 60 | 52 | 63 | 62 | 105 | 121 | 81 | 115 |
| 1732 | 1663 | 1832 | 1531 | 1027 | 900 | 1211 | 1004 | 569 | 567 | 618 | 578 | 66 | 61 | 64 | 59 | 74 | 113 | 97 | 90 |
| 1668 | 1628 | 1664 | 1666 | 914 | 795 | 1093 | 1142 | 590 | 519 | 599 | 552 | 69 | 83 | 61 | 58 | 103 | 112 | 93 | 86 |
| 1789 | 1800 | 1697 | 1637 | 1063 | 1044 | 1107 | 1202 | 608 | 624 | 624 | 557 | 75 | 73 | 56 | 62 | 115 | 96 | 109 | 108 |
| 1648 | 1820 | 1551 | 1795 | 914 | 892 | 1235 | 1025 | 543 | 601 | 594 | 600 | 70 | 65 | 65 | 56 | 94 | 85 | 88 | 81 |
| 1630 | 1716 | 1772 | 1855 | 1010 | 1210 | 1088 | 824 | 600 | 608 | 594 | 604 | 70 | 70 | 70 | 67 | 80 | 89 | 116 | 81 |
| 1939 | 1797 | 1888 | 1691 | 947 | 926 | 902 | 1015 | 572 | 584 | 589 | 578 | 77 | 77 | 69 | 54 | 90 | 92 | 103 | 103 |
| 1841 | 1705 | 1843 | 1853 | 889 | 1038 | 898 | 1031 | 649 | 599 | 606 | 595 | 49 | 57 | 75 | 66 | 83 | 91 | 115 | 77 |
| 1948 | 1727 | 1813 | 1675 | 1002 | 1042 | 951 | 909 | 555 | 593 | 540 | 631 | 58 | 74 | 68 | 72 | 103 | 75 | 85 | 83 |
| 1935 | 1636 | 1596 | 1814 | 946 | 971 | 1082 | 1044 | 613 | 554 | 577 | 592 | 51 | 71 | 59 | 69 | 96 | 116 | 94 | 86 |
| 1801 | 1771 | 1619 | 1659 | 1071 | 793 | 1080 | 1009 | 570 | 597 | 584 | 540 | 53 | 63 | 62 | 57 | 81 | 63 | 90 | 89 |
| 1797 | 1801 | 1697 | 1639 | 1145 | 893 | 938 | 818 | 581 | 595 | 584 | 564 | 61 | 71 | 65 | 58 | 79 | 106 | 88 | 102 |
| 1893 | 1703 | 1665 | 1732 | 1035 | 1049 | 991 | 1133 | 644 | 641 | 580 | 556 | 63 | 70 | 75 | 53 | 85 | 79 | 117 | 93 |
| 1663 | 1700 | 1876 | 1646 | 982 | 882 | 893 | 996 | 607 | 572 | 548 | 589 | 74 | 57 | 72 | 58 | 90 | 87 | 95 | 78 |
| 1659 | 1847 | 1880 | 1538 | 1045 | 958 | 1016 | 1022 | 520 | 577 | 621 | 582 | 73 | 64 | 63 | 79 | 97 | 103 | 108 | 96 |
| 1673 | 1838 | 1640 | 1664 | 1025 | 1166 | 1146 | 994 | 560 | 588 | 571 | 564 | 60 | 59 | 59 | 59 | 108 | 103 | 75 | 97 |
| 1841 | 1723 | 1605 | 1704 | 851 | 1251 | 895 | 1061 | 609 | 535 | 583 | 579 | 64 | 62 | 65 | 68 | 107 | 73 | 103 | 124 |
| 1821 | 1858 | 1710 | 1731 | 811 | 927 | 950 | 898 | 627 | 525 | 592 | 610 | 65 | 66 | 54 | 58 | 90 | 84 | 97 | 103 |
| 2009 | 1708 | 1645 | 1582 | 1021 | 897 | 936 | 1041 | 614 | 584 | 610 | 568 | 59 | 63 | 64 | 69 | 91 | 82 | 78 | 101 |
| 1768 | 1500 | 1642 | 1814 | 765 | 1242 | 1010 | 1276 | 587 | 541 | 573 | 551 | 63 | 68 | 75 | 62 | 98 | 94 | 85 | 94 |
| 1944 | 1958 | 1959 | 1630 | 865 | 900 | 999 | 975 | 635 | 591 | 614 | 586 | 81 | 58 | 60 | 61 | 81 | 87 | 100 | 89 |
| 1571 | 1805 | 1531 | 1791 | 1085 | 981 | 873 | 1059 | 571 | 543 | 580 | 632 | 70 | 60 | 58 | 71 | 102 | 104 | 103 | 99 |
| 1614 | 1739 | 1476 | 1509 | 1192 | 890 | 910 | 886 | 578 | 534 | 586 | 577 | 60 | 82 | 63 | 82 | 105 | 89 | 82 | 94 |
| 1678 | 1749 | 1781 | 1697 | 982 | 944 | 1170 | 1163 | 569 | 561 | 558 | 652 | 56 | 68 | 77 | 74 | 107 | 92 | 107 | 114 |
| 1538 | 1745 | 1808 | 1774 | 868 | 1120 | 1037 | 1008 | 571 | 651 | 588 | 597 | 58 | 62 | 55 | 65 | 90 | 94 | 78 | 82 |
| 1796 | 1782 | 1825 | 1687 | 1087 | 1155 | 922 | 1104 | 660 | 574 | 617 | 618 | 68 | 68 | 64 | 75 | 110 | 80 | 101 | 85 |
| 1467 | 1942 | 1677 | 1527 | 1032 | 885 | 1095 | 1151 | 599 | 590 | 542 | 529 | 55 | 72 | 65 | 65 | 92 | 88 | 111 | 90 |
| 1825 | 1855 | 1577 | 1476 | 965 | 1195 | 1069 | 1106 | 567 | 616 | 604 | 600 | 62 | 59 | 63 | 51 | 112 | 97 | 81 | 119 |
| 2058 | 1762 | 1573 | 1815 | 1003 | 1022 | 1189 | 1068 | 555 | 601 | 596 | 584 | 80 | 67 | 63 | 60 | 81 | 105 | 94 | 112 |
| 1955 | 2073 | 1986 | 1679 | 936 | 1028 | 869 | 954 | 624 | 607 | 571 | 557 | 51 | 55 | 61 | 67 | 106 | 89 | 86 | 84 |
| 1743 | 1724 | 1638 | 1832 | 920 | 1102 | 1175 | 865 | 614 | 609 | 570 | 578 | 71 | 66 | 59 | 65 | 100 | 123 | 109 | 98 |
| 1714 | 1694 | 1802 | 1680 | 940 | 1164 | 1227 | 1073 | 594 | 587 | 556 | 583 | 66 | 64 | 74 | 62 | 99 | 107 | 131 | 85 |
| 1914 | 1649 | 1751 | 1775 | 902 | 1093 | 1176 | 939 | 614 | 556 | 560 | 569 | 60 | 62 | 85 | 54 | 85 | 99 | 91 | 72 |
| 1991 | 1819 | 1711 | 1785 | 946 | 1124 | 894 | 846 | 546 | 587 | 627 | 586 | 68 | 67 | 72 | 58 | 91 | 118 | 102 | 84 |
| 1709 | 1757 | 1760 | 1725 | 924 | 1161 | 1334 | 932 | 539 | 580 | 571 | 582 | 59 | 80 | 54 | 62 | 90 | 88 | 84 | 107 |
| 1762 | 1688 | 1762 | 1860 | 1258 | 921 | 1134 | 774 | 553 | 563 | 537 | 511 | 59 | 75 | 48 | 62 | 73 | 85 | 103 | 116 |
| 1729 | 1438 | 2089 | 1645 | 1148 | 975 | 970 | 1083 | 581 | 568 | 643 | 553 | 66 | 81 | 67 | 69 | 89 | 100 | 122 | 84 |
| 1748 | 1641 | 1795 | 1660 | 1045 | 967 | 835 | 1216 | 605 | 610 | 544 | 550 | 63 | 64 | 64 | 63 | 80 | 108 | 93 | 81 |
| 1694 | 1726 | 1822 | 1851 | 1038 | 822 | 844 | 939 | 506 | 633 | 583 | 586 | 67 | 60 | 63 | 55 | 78 | 119 | 96 | 109 |
| 1625 | 1739 | 1577 | 1680 | 1045 | 1009 | 901 | 929 | 573 | 589 | 575 | 564 | 60 | 59 | 72 | 73 | 71 | 99 | 94 | 98 |
| 1695 | 1782 | 1580 | 1740 | 1159 | 1010 | 815 | 1113 | 611 | 635 | 538 | 602 | 64 | 65 | 61 | 70 | 98 | 100 | 79 | 107 |
| 1826 | 1695 | 1654 | 1693 | 917 | 1044 | 899 | 989 | 526 | 599 | 561 | 577 | 55 | 54 | 56 | 66 | 104 | 69 | 115 | 116 |
| 1730 | 1866 | 1649 | 1726 | 1074 | 1079 | 1166 | 1150 | 652 | 585 | 570 | 620 | 63 | 76 | 54 | 69 | 91 | 106 | 97 | 108 |
| 1702 | 1719 | 1921 | 1682 | 1195 | 975 | 1020 | 1014 | 597 | 547 | 538 | 555 | 84 | 72 | 63 | 67 | 85 | 103 | 90 | 86 |
| 1805 | 1800 | 1968 | 1586 | 960 | 874 | 955 | 967 | 610 | 584 | 558 | 572 | 56 | 67 | 59 | 59 | 90 | 91 | 82 | 106 |
| 1791 | 1722 | 1756 | 1689 | 972 | 961 | 1037 | 1081 | 564 | 561 | 602 | 647 | 67 | 69 | 67 | 68 | 83 | 104 | 109 | 97 |
| 1822 | 1953 | 2096 | 1896 | 842 | 1079 | 929 | 1005 | 570 | 534 | 604 | 603 | 60 | 54 | 59 | 72 | 77 | 95 | 80 | 99 |
| 2109 | 1962 | 1840 | 1801 | 1103 | 1020 | 924 | 1002 | 624 | 529 | 616 | 611 | 60 | 67 | 69 | 53 | 70 | 86 | 76 | 91 |
| 2003 | 1912 | 1584 | 1839 | 890 | 994 | 932 | 1063 | 632 | 610 | 589 | 594 | 63 | 63 | 66 | 62 | 114 | 96 | 88 | 83 |
| 1864 | 1581 | 1815 | 1629 | 1238 | 1089 | 979 | 1093 | 695 | 584 | 577 | 577 | 77 | 72 | 55 | 78 | 82 | 88 | 80 | 99 |
| 1927 | 1820 | 1846 | 1554 | 1076 | 847 | 959 | 1077 | 591 | 598 | 600 | 587 | 57 | 53 | 63 | 77 | 91 | 95 | 95 | 94 |
| 1743 | 1872 | 1665 | 1631 | 1071 | 968 | 951 | 1026 | 572 | 551 | 615 | 624 | 52 | 56 | 67 | 69 | 98 | 75 | 84 | 98 |
| 1732 | 1817 | 1810 | 1627 | 1028 | 979 | 778 | 1186 | 632 | 551 | 576 | 595 | 66 | 63 | 62 | 57 | 107 | 96 | 110 | 106 |
| 1692 | 1578 | 1807 | 1569 | 881 | 1100 | 962 | 1192 | 643 | 536 | 543 | 561 | 64 | 77 | 72 | 63 | 95 | 115 | 96 | 101 |
| 1757 | 1982 | 1887 | 1645 | 993 | 916 | 912 | 1111 | 539 | 605 | 610 | 545 | 70 | 57 | 60 | 60 | 113 | 97 | 94 | 113 |
| 1930 | 1834 | 1823 | 1839 | 883 | 1048 | 907 | 882 | 651 | 608 | 561 | 579 | 52 | 62 | 68 | 55 | 106 | 76 | 111 | 79 |
| 1681 | 1822 | 1943 | 1690 | 1017 | 1030 | 920 | 1147 | 537 | 578 | 567 | 545 | 63 | 61 | 73 | 68 | 94 | 131 | 87 | 96 |
| 1686 | 1553 | 1746 | 1674 | 859 | 996 | 862 | 1085 | 584 | 586 | 585 | 589 | 58 | 60 | 58 | 56 | 114 | 90 | 89 | 89 |
| 1634 | 1682 | 1716 | 1933 | 1065 | 1024 | 967 | 981 | 577 | 629 | 613 | 640 | 64 | 55 | 75 | 67 | 103 | 97 | 103 | 83 |
| 1605 | 1521 | 1752 | 1769 | 956 | 853 | 907 | 897 | 627 | 559 | 588 | 583 | 55 | 60 | 68 | 62 | 111 | 103 | 91 | 89 |
| 1742 | 1704 | 1815 | 1826 | 1041 | 1078 | 1121 | 1057 | 591 | 673 | 606 | 532 | 59 | 62 | 58 | 68 | 98 | 83 | 118 | 89 |
| 1925 | 1926 | 1707 | 1567 | 933 | 859 | 872 | 835 | 581 | 628 | 592 | 527 | 54 | 69 | 77 | 64 | 106 | 96 | 92 | 101 |
| 1502 | 1697 | 1695 | 1636 | 853 | 919 | 888 | 925 | 594 | 589 | 616 | 588 | 51 | 72 | 77 | 63 | 92 | 116 | 86 | 78 |
| 1818 | 1941 | 2028 | 1852 | 989 | 1256 | 1030 | 967 | 591 | 547 | 593 | 622 | 69 | 59 | 57 | 61 | 110 | 86 | 105 | 105 |
| 1966 | 1732 | 1811 | 1719 | 1019 | 1030 | 1001 | 819 | 560 | 605 | 616 | 600 | 68 | 53 | 60 | 57 | 97 | 115 | 107 | 73 |
| 1661 | 1827 | 1799 | 1592 | 821 | 796 | 881 | 1040 | 566 | 553 | 583 | 579 | 58 | 65 | 62 | 65 | 102 | 119 | 94 | 103 |
| 1867 | 1628 | 1840 | 1699 | 761 | 1066 | 1054 | 975 | 633 | 587 | 626 | 566 | 69 | 61 | 68 | 69 | 98 | 121 | 109 | 79 |
| 1660 | 1751 | 1731 | 2002 | 846 | 868 | 1001 | 965 | 587 | 531 | 587 | 513 | 80 | 51 | 50 | 73 | 110 | 90 | 85 | 78 |
| 2074 | 1920 | 1913 | 1898 | 979 | 979 | 889 | 982 | 616 | 603 | 565 | 592 | 63 | 66 | 63 | 67 | 93 | 118 | 105 | 108 |
| 1862 | 1727 | 1713 | 1803 | 829 | 1522 | 1064 | 816 | 533 | 635 | 590 | 583 | 61 | 64 | 56 | 62 | 111 | 94 | 90 | 87 |
| 1750 | 1794 | 1746 | 1599 | 1138 | 1062 | 995 | 968 | 616 | 619 | 636 | 545 | 64 | 68 | 66 | 70 | 83 | 89 | 84 | 96 |
| 1913 | 1861 | 1792 | 1601 | 861 | 915 | 1121 | 898 | 537 | 541 | 601 | 621 | 56 | 55 | 71 | 64 | 86 | 108 | 79 | 96 |

**Bibliography**

Abdi D, Cade-Menun B J, Ziadi N and Parent L o-Ã t 2014 Long-term impact of tillage practices and phosphorus fertilization on soil phosphorus forms as determined by P nuclear magnetic resonance spectroscopy. J. Environ. Qual. 43, 1431-1441.

Ahlgren J, Djodjic F, Borjesson G and Mattsson L 2013 Identification and quantification of organic phosphorus forms in soils from fertility experiments. Soil use Manage. 29, 24-35.

Annaheim K, Doolette A, Smernik R, Mayer J, Oberson A, Frossard E and Bunemann E 2015 Long-term addition of organic fertilizers has little effect on soil organic phosphorus as characterized by 31 P NMR spectroscopy and enzyme additions. Geoderma 257, 67-77.

Bourke D, Dowding P, Tunney H, O'Brien J E and Jeffrey D W 2008 The organic phosphorus composition of an Irish grassland soil. Proc. R. Ir. Acad. 108 17-28.

Bunemann E, Marschner P, Smernik R, Conyers M and McNeill A 2008 Soil organic phosphorus and microbial community composition as affected by 26 years of different management strategies. Biol. Fertil. Soils 44, 717-726.

Cade-Menun B J, Carter M R, James D C and Liu C W 2010 Phosphorus forms and chemistry in the soil profile under long-term conservation tillage: A phosphorus-31 nuclear magnetic resonance study. J. Environ. Qual. 39, 1647-1656.

Chapuis-Lardy L, Brossard M and Quiquampoix H 2001 Assessing organic phosphorus status of Cerrado oxisols (Brazil) using 31P-NMR spectroscopy and phosphomonoesterase activity measurement. Can. J. Soil Sci. 81, 591-601.

Condron L, Frossard E, Tiessen H, Newmans R and Stewart J 1990 Chemical nature of organic phosphorus in cultivated and uncultivated soils under different environmental conditions. Eur. J. Soil Sci. 41, 41-50.

Doolette A, Smernik R and Dougherty W 2009 Spiking improved solution phosphorus-31 nuclear magnetic resonance identification of soil phosphorus compounds. Soil Sci. Soc. Am. J. 73, 919-927.

Doolette A, Smernik R and Dougherty W 2011 A quantitative assessment of phosphorus forms in some Australian soils. Soil Res. 49, 152-165.

Dougherty W J, Smernik R J, Bunemann E K and Chittleborough D J 2007 On the use of hydrofluoric acid pretreatment of soils for phosphorus-31 nuclear magnetic resonance analyses. Soil Sci. Soc. Am. J. 71, 1111-1118.

Gatiboni L C, Rheinheimer D d S, Flores A F C, Anghinoni I, Kaminski J and Lima M A S 2007 Phosphorus Forms and Availability Assessed by 31P-NMR in Successively Cropped Soil. Commun. Soil Sci. Plant Anal. 36, 2625-2640.

George T, Turner B, Gregory P, Cade-Menun B and Richardson A 2006 Depletion of organic phosphorus from Oxisols in relation to phosphatase activities in the rhizosphere. Eur. J. Soil Sci. 57, 47-57.

Giles C D, Cade-Menun B J, Liu C W and Hill J E 2015 The short-term transport and transformation of phosphorus species in a saturated soil following poultry manure amendment and leaching. Geoderma 257, 134-141.

Guggenberger G, Christensen B T, Rubaek G and Zech W 1996a Land-use and fertilization effects on P forms in two European soils: resin extraction and 31P-NMR analysis. Eur. J. Soil Sci. 47, 605-614.

Guggenberger G, Haumaier L, Zech W and Thomas R 1996b Assessing the organic phosphorus status of an Oxisol under tropical pastures following native savanna using 31 P NMR spectroscopy. Biol. Fertil. Soils 23, 332-339.

Hill J E and Cade-Menun B J 2009 Phosphorus-31 nuclear magnetic resonance spectroscopy transect study of poultry operations on the Delmarva Peninsula. J. Environ. Qual. 38, 130-138.

Jin Y, Liang X, He M, Liu Y, Tian G and Shi J 2016 Manure biochar influence upon soil properties, phosphorus distribution and phosphatase activities: a microcosm incubation study. Chemosphere 142, 128-135.

Koopmans G, Chardon W, Dolfing J, Oenema O, Van der Meer P and Van Riemsdijk W 2003 Wet chemical and phosphorus-31 nuclear magnetic resonance analysis of phosphorus speciation in a sandy soil receiving long-term fertilizer or animal manure applications. J. Environ. Qual. 32, 287-295.

Lehmann J, Lan Z, Hyland C, Sato S, Solomon D and Ketterings Q M 2005 Long-term dynamics of phosphorus forms and retention in manure-amended soils. Environmental Science & Technology 39, 6672-6680.

Leinweber P, Haumaier L and Zech W 1997 Sequential extractions and 31P-NMR spectroscopy of phosphorus forms in animal manures, whole soils and particle-size separates from a densely populated livestock area in northwest Germany. Biol. Fertil. Soils 25, 89-94.

Liu J, Hu Y, Yang J, Abdi D and Cade-Menun B J 2014 Investigation of soil legacy phosphorus transformation in long-term agricultural fields using sequential fractionation, P K-edge XANES and solution P NMR spectroscopy. Environ. Sci. Technol. 49, 168-176.

McDowell R, Condron L, Stewart I and Cave V 2005 Chemical nature and diversity of phosphorus in New Zealand pasture soils using 31P nuclear magnetic resonance spectroscopy and sequential fractionation. Nutr. Cycling Agroecosyst. 72, 241-254.

McDowell R and Koopmans G 2006 Assessing the bioavailability of dissolved organic phosphorus in pasture and cultivated soils treated with different rates of nitrogen fertiliser. Soil Biol. Biochem. 38, 61-70.

McDowell R and Stewart I 2006 The phosphorus composition of contrasting soils in pastoral, native and forest management in Otago, New Zealand: sequential extraction and 31 P NMR. Geoderma 130, 176-189.

McLaren T I, Smernik R J, Guppy C N, Bell M J and Tighe M K 2014 The Organic P Composition of Vertisols as Determined by P NMR Spectroscopy. Soil Sci. Soc. Am. J. 78, 1893-1902.

Moller A, Kaiser K, Amelung W, Niamskul C, Udomsri S, Puthawong M, Haumaier L and Zech W 2000 Forms of organic C and P extracted from tropical soils as assessed by liquid-state 13C-and 31P-NMR spectroscopy. Soil Res. 38, 1017-1036.

Murphy P, Bell A and Turner B 2009 Phosphorus speciation in temperate basaltic grassland soils by solution 31P NMR spectroscopy. Eur. J. Soil Sci. 60, 638-651.

Soinne H, Uusitalo R, Sarvi M, Turtola E and Hartikainen H 2011 Characterization of soil phosphorus in differently managed clay soil by chemical extraction methods and 31P NMR spectroscopy. Commun. Soil Sci. Plant Anal. 42, 1995-2011.

Solomon D and Lehman N 2000 Loss of phosphorus from soil in semi-arid northern Tanzania as a result of cropping: evidence from sequential extraction and 31P-NMR spectroscopy. Eur. J. Soil Sci. 51, 699-708.

Solomon D, Lehmann J, Mamo T, Fritzsche F and Zech W 2002 Phosphorus forms and dynamics as influenced by land use changes in the sub-humid Ethiopian highlands. Geoderma 105, 21-48.

Stutter M I, Shand C A, George T S, Blackwell M S, Dixon L, Bol R, MacKay R L, Richardson A E, Condron L M and Haygarth P M 2015 Land use and soil factors affecting accumulation of phosphorus species in temperate soils. Geoderma 257, 29-39.

Turner B L 2006 Organic phosphorus in Madagascan rice soils. Geoderma 136, 279-288.

Turner B L, Cade-Menun B J and Westermann D T 2003a Organic phosphorus composition and potential bioavailability in semi-arid arable soils of the western United States. Soil Sci. Soc. Am. J. 67, 1168-1179.

Turner B L, Mahieu N and Condron L M 2003b The phosphorus composition of temperate pasture soils determined by NaOH–EDTA extraction and solution 31P NMR spectroscopy. Org. Geochem. 34, 1199-1210.
